# Extensions of multinomial processing tree models for continuous variables: A simulation study comparing parametric and non-parametric approaches

**DOI:** 10.3758/s13428-025-02896-9

**Published:** 2025-12-08

**Authors:** Anahí Gutkin, Daniel W. Heck

**Affiliations:** 1 Faculty of Medicine, Universidad Francisco de Vitoria, Madrid, Spain; 2 Department of Psychology, Philipps-Universität Marburg, Marburg, Germany

**Keywords:** Cognitive modeling, Non-parametric approaches, MPT models, Response times

## Abstract

Both parametric and non-parametric extensions of the multinomial processing tree (MPT) models have been proposed for jointly modeling discrete and continuous variables. Since the two approaches have not yet been compared systematically, we assess their power and robustness in three simulation studies focusing on the weapon identification task. In this context, two statistically equivalent MPT models have been proposed, namely, the preemptive-conflict-resolution model (PCRM) and the default-interventionist model (DIM), which differ only in their assumptions regarding the order of latent processes (i.e., response times, RTs). The first simulation evaluates the calibration and statistical power of the nonstandard goodness-of-fit test for the parametric approach (i.e., the Dzhaparidze–Nikulin statistic), as well as the ability of different distributional assumptions to fit simulated RT data. The second simulation compares nested models to study the power for testing hypotheses about RTs within each model. The third simulation focuses on model-recovery performance for the two non-nested models. In all three simulations, we manipulated the size and nature of discrepancies (location/scale or shape) between latent RT distributions, sample size, and parametric assumptions. Results show that the parametric approach has higher statistical power but is also sensitive to misspecifications of distributional assumptions. In contrast, the non-parametric approach is more robust but less powerful, especially with small samples. We provide recommendations on when to use each approach and highlight the importance of properly specifying and selecting extended MPT models.

**Supplementary Information:**

The online version contains supplementary material available at 10.3758/s13428-025-02896-9.

## Introduction

Multinomial processing tree (MPT) models have been shown to be useful in several areas of psychology, as they enable studying the validity of theories that allow the same responses or behavior to be caused by different cognitive processes (Batchelder & Riefer, 1999; Erdfelder et al., 2009; Hütter & Klauer, 2016). MPT models were originally developed as stochastic measurement models for discrete data, but have recently been extended for jointly modeling discrete and continuous variables such as response times (RTs; Heck et al., 2018; Heck & Erdfelder, 2016; Klauer & Kellen, 2018). The approaches build on the method proposed by Hu (2001), which is a generalization of Link's (1982) model.

Modeling discrete responses and RTs jointly within RT-extended MPT models presents several advantages over handling categorical and continuous data separately (Gutkin et al., 2024). Analyzing a single variable in isolation can increase type I errors, where researchers report a significant effect in one metric (e.g., accuracy) while ignoring nonsignificant results in another (e.g., RTs), leading to false positives. Separate analyses can also result in a loss of information and statistical power, as performance differences may affect both variables jointly while not producing a significant effect in either variable. Additionally, it is more difficult to detect dependencies of discrete responses and RTs, such as the speed–accuracy trade-off, making overall performance harder to interpret (Liesefeld & Janczyk, 2019; Voss et al., 2013). These challenges can be addressed by fitting models that jointly account for continuous and discrete variables. One of the most significant advantages of RT-extended MPT models is that they enable testing hypotheses about continuous variables that are not directly observable. As such, they can be used to validate the assumptions of an MPT model concerning the distribution of RTs linked to its branches. Moreover, RT-extended MPT models allow researchers to select between competing models that can be distinguished only by considering their predictions for RTs. To illustrate the benefits of RT-extended MPT in model selection, we will first explain the specific experimental paradigm and substantive research question that will serve as a running example in the present study.

In the weapon identification task (Payne, 2001; Rivers, 2017), two images are briefly displayed one after another on a monitor. Participants are instructed to disregard the first image of a face, which is portrayed to be merely a location marker for the second image. In fact, the first image serves as a racial prime and displays either a white or a black face, while the second image shows either a gun or a tool. Participants are asked to identify whether the second image depicts a tool or a gun. To explain stereotype processing in the weapon identification task, researchers have applied an MPT model known as the process dissociation procedure model (Jacoby, 1991). The model aims to disentangle the contributions of controlled and automatic processes on task performance by estimating the probability of each process (automatic and controlled) for different racial primes. However, the model does not make testable assumptions about the specific order of the hypothesized processes. According to Klauer & Voss (2008), different assumptions about the order of latent processes are compatible with the process dissociation procedure model. Even though all these models include both automatic and controlled processes, they differ in the structure of conditional probabilities, the temporal sequence, and the nature of hypothesized processes. Among these models, we can distinguish the preemptive-conflict-resolution model (PCRM) and the default-interventionist model (DIM).

**Fig. 1:**
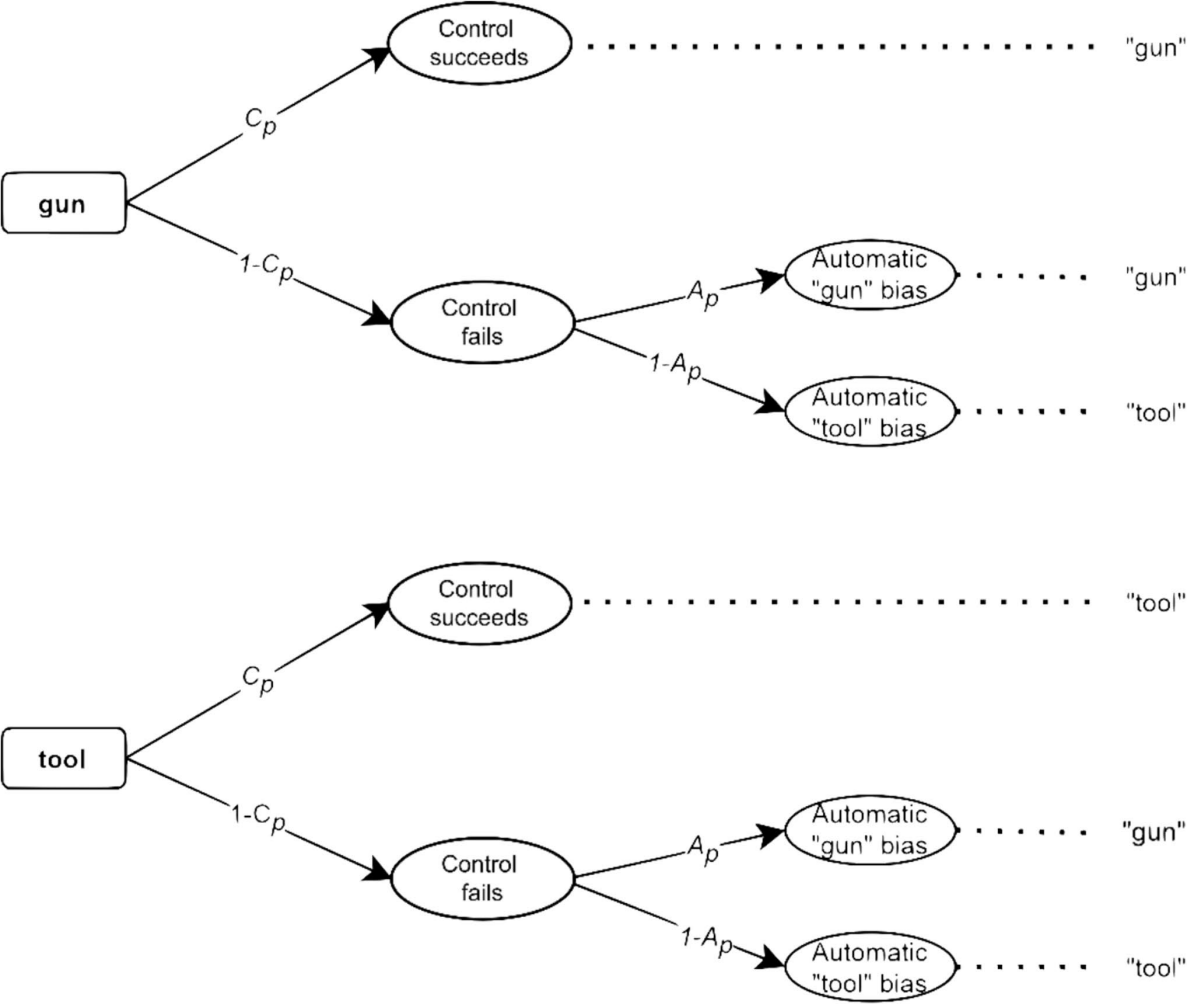
Classic preemptive-conflict-resolution model (PCRM). *Note*. The figure only shows the trees for one type of racial prime. To obtain the full MPT model, it is necessary to create two separate gun and tool trees for each racial prime (gun-black, tool-black, gun-white, tool-white) and replace the subscript *p* by *w* for white and *b* for black racial primes, respectively. Overall, the model has the following four probability parameters: *C*_*b*_ (controlled process probability for black prime), *C*_*w*_ (controlled process probability for white prime), *A*_*b*_ (automatic process bias probability for black prime), and *A*_*w*_ (automatic process bias probability for white prime). The last column on the right represents the observed response (“gun” or “tool”) associated with each path of the MPT model. Given the observed responses and presented stimuli (gun or tool), four categorical response types can be identified: *hit* (gun presented, correct "gun" response), *miss* (gun presented, "tool" response), *correct rejection* (tool presented, correct "tool" response), and *false alarm* (tool presented, "gun" response). These categories are used in the model files provided in the supplementary material.) associated with each path of the MPT model

The original version of the PCRM (Evans, 2007) includes only one controlled and one automatic process. Figure 1 shows that controlled processing of the presented target image occurs with probability $C$, leading to the correct identification of a gun or tool. Conversely, with probability $1-C$, the controlled process fails which in turn triggers an automatic response. The parameter $A$ represents the conditional probability that the automatic process results in a “gun” response, while the probability $1-A$ represents the probability of a “tool” response. Importantly, Fig. 1 shows a simplified version of the actual MPT model, as it does not indicate that the $A$ and $C$ parameters can vary depending on the presented racial prime. To denote this, we use a subscript *p,* which needs to be replaced by the index *b* for black racial primes or *w* for white racial primes. The PCRM assumes that the controlled process precedes and dominates the automatic process. This means that the controlled process initially allows discriminating between images of guns and tools, and only when this process is not successful, the automatic process determines the response.

**Fig. 2:**
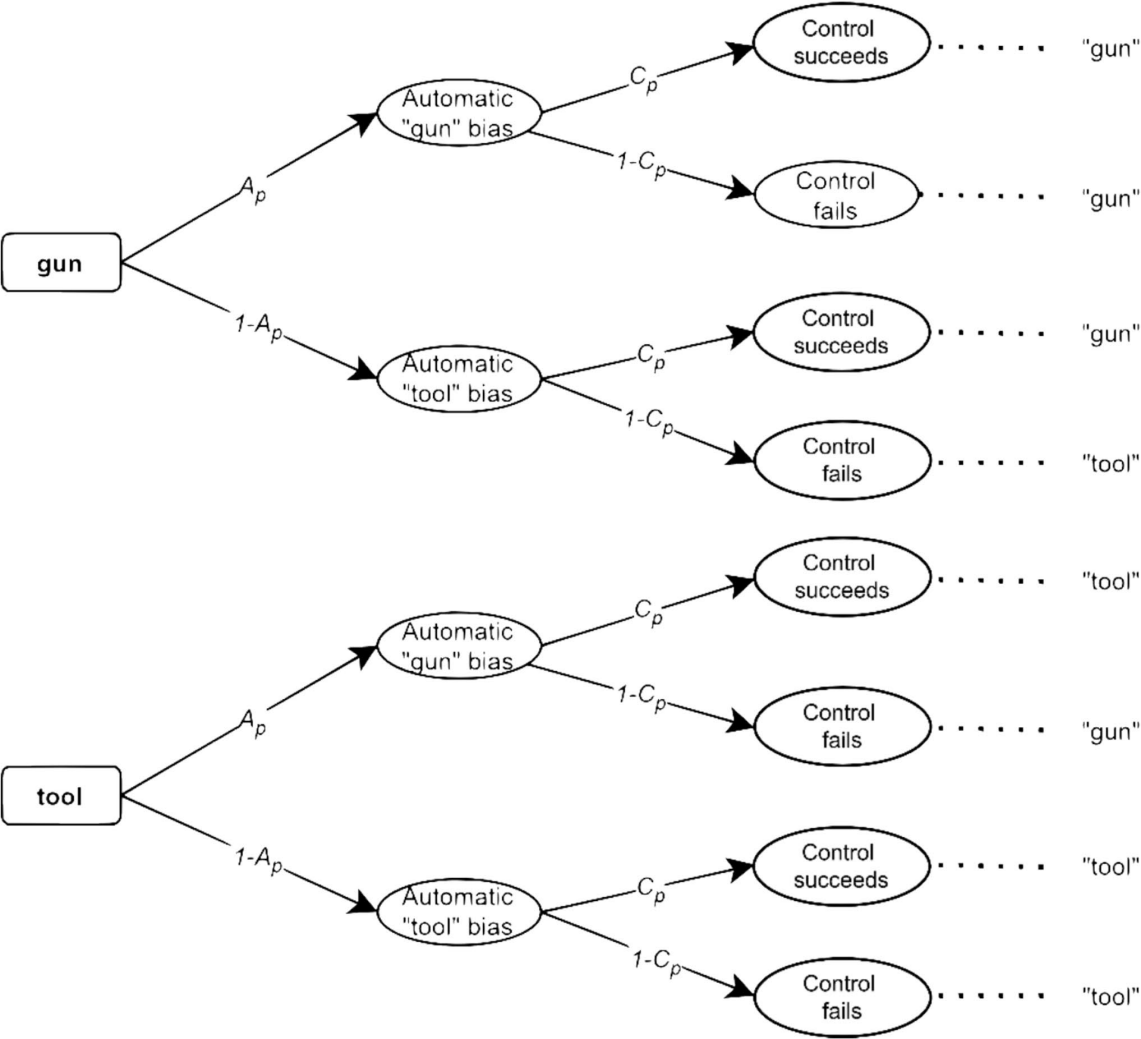
Classic default-interventionist model (DIM). *Note.* The probability of the automatic, *A*, and controlled, , process vary according to the racial prime *p* = {*b* = black, *w* = white}, which is not presented in the figure. The last column on the right represents the resulting response categories for each path of the MPT model.

An alternative model for the process dissociation procedure is the DIM (Evans, 2007). As depicted in Fig. 2, it suggests that an automatic bias first triggers a default response, which can sometimes be modified by a subsequent intervention through a successful controlled process (Evans, 2007). Thus, there is an initial, automatic processing stage in which racial associations are activated (e.g., the association of “black” and “gun”). Respondents are assumed to give a "gun" response with probability $A$ or a "tool" response with probability $1-A$. After the activation of the default racial association, a controlled process may or may not occur with the conditional probability $C$ or $1-C$, respectively. The controlled process represents an attempt to overcome the prior bias (Klauer & Voss, 2008), where the probability of each process being activated depends on the type of racial prime presented (i.e., subscript *p* should again be substituted by *b* or *w* for black or white racial primes, respectively).

The two models involve different theoretical assumptions about the nature of the latent processes involved. Nevertheless, they are statistically equivalent (Evans, 2007; Laukenmann et al., 2023). This means that, despite their different interpretation and graphical representations as probability trees, both models are algebraically identical, yield the same predictions, and produce the same model fit in terms of the log-likelihood (for a mathematical proof of this equivalence, see Laukenmann et al., 2023). As a result, it is not possible to test the two competing models against each other based solely on response frequencies. Apart from substantive and methodological implications, this limitation has important practical consequences as each of the models suggests different bias-reduction interventions (Calanchini et al., 2021; Gonsalkorale et al., 2011). To facilitate an empirical comparison of the two MPT models, it is necessary to extend them with RTs. By using RT-extended MPT models to include RTs, it is possible to assess whether the observed data more closely match the predictions of the DIM or the PCRM. By testing the validity of the models, it may be possible in the future to select the most appropriate intervention to reduce the negative consequences of stereotype processing.

**Fig. 3:**
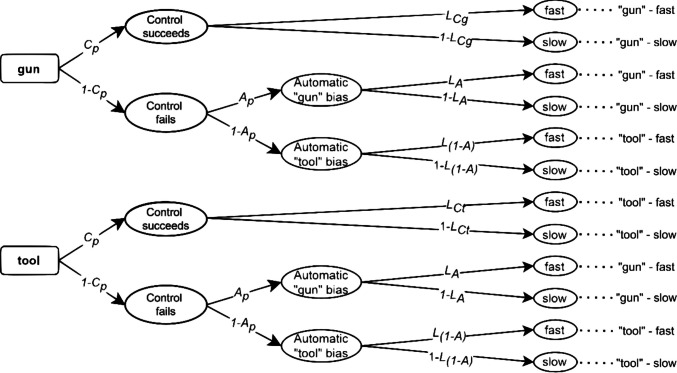
RT-Extended preemptive-conflict-resolution model (PCRM) with response times. *Note.* The automatic and controlled process probabilities *A* and *C* vary according to the racial prime* p* = {*b* = black, *w* = white}. The *L* parameters are defined as the probability of a fast response and can vary according to the process that triggered them, as indicated by the subscripts *C*, *A*, and *1-A* and the stimulus presented as indicated by the subscripts *g* = gun and* t* = tool.

**Fig. 4:**
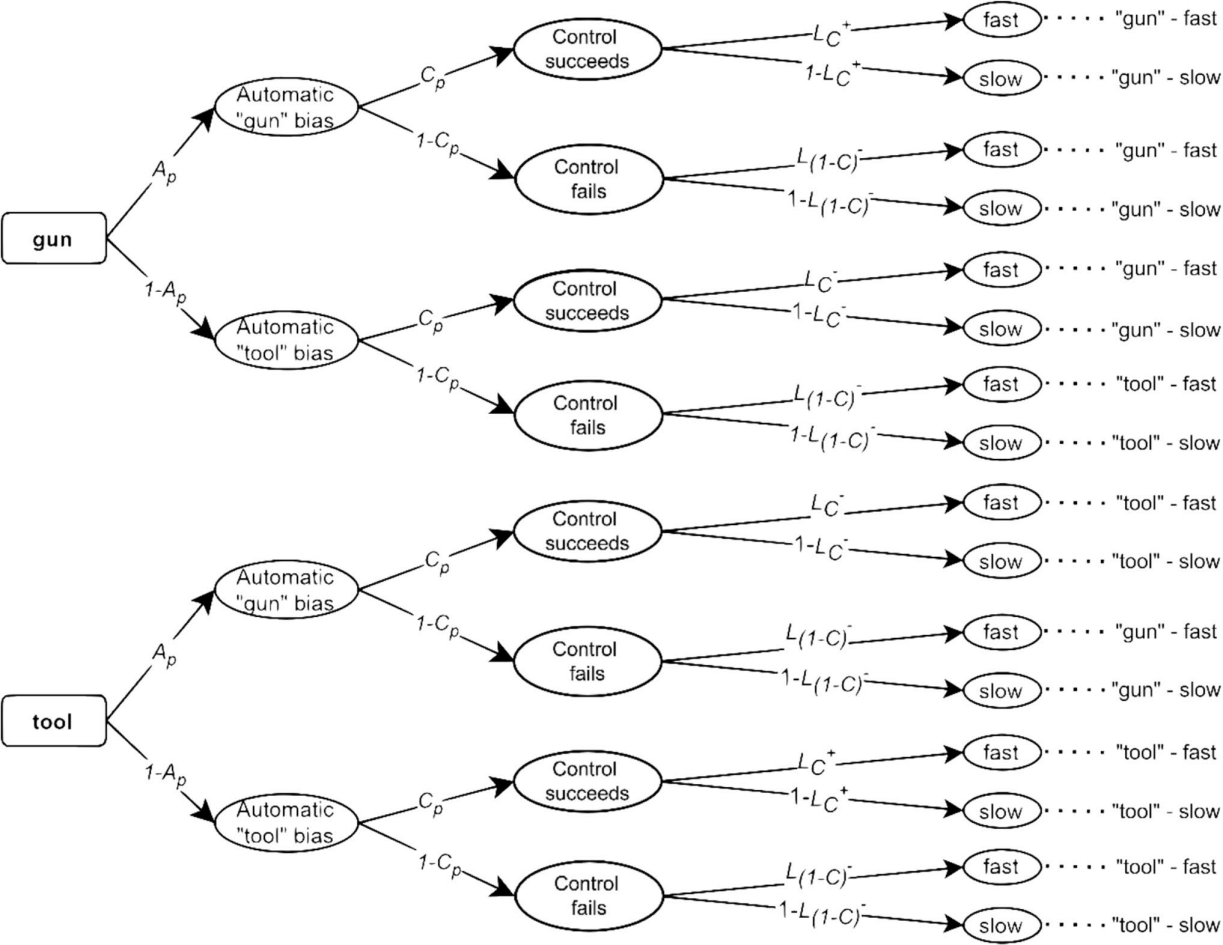
RT-Extended default-interventionist model (DIM) with response times. *Note.* The automatic, *A*, and controlled, *C*, process probabilities vary according to the racial prime *p* = {*b* =black, *w* = white}. The *L* parameters can vary according to the process that triggered them (subscripts *C* or *1-C*). Additionally, the controlled process latency parameter depends on whether overcoming the default association is congruent (+) or incongruent (–) with the target

To explain how to include RT predictions in the above models, we first need to choose a method for including continuous variables in MPT models. Here, we focus on different methods to do so: a non-parametric (Heck & Erdfelder, 2016) and a parametric approach (Heck et al., 2018; Klauer & Kellen al., 2018; Klauer et al., 2024). The former was proposed by Heck and Erdfelder (2016) and transforms the continuous variable into an ordinal one. The original MPT model is transformed into an extended MPT model by dividing all branches of the MPT model into $B$ subbranches. Each extended branch corresponds to a specific bin of an RT histogram (Van Zandt, 2000). The probability of each bin can be estimated using standard software for MPT modeling (for a tutorial, see Schmidt et al., 2023). For example, if we categorize RTs into two bins ($B=2$, resulting in more fine-grained “fast” and “slow” response categories), we can estimate the branch probabilities that a latent process results in a relatively fast or a relatively slow response^1^. As these are complementary probabilities, we can associate them with the latency parameters $L$ and $1-L$, respectively.^2^ In Figs. 3 and 4, we show the two-bin extension of the PCRM and the DIM, respectively. To distinguish the probability of fast responding for different branches of an MPT model, a subscript is added to each $L$ parameter to refer to the corresponding latent processes.

In Fig. 3, the PCRM has been extended to include the latency parameters ${L}_{Cg}$, ${L}_{Ct}$, ${L}_{A}$, and ${L}_{(1-A)}$. These parameters represent the conditional probabilities of giving a fast response when being in the controlled state (separately for images of guns and tools; ${L}_{Cg}$ and ${L}_{Ct}$) or when being in the fast automatic state (${L}_{A}$ and ${L}_{(1-A)}$). The model assumes that automatic processing latencies result from an automatic activation upon a failure of the control process. While this implies that errors should not be faster than correct responses (Klauer & Voss, 2008), there are no strong reasons to believe that the automatic activation towards *tool* is faster or slower than the automatic activation towards *gun*; therefore

$${L}_{A}={L}_{(1-A)}$$

Controlled responding is more costly and requires more time than automatic responding. Hence, for trials showing tools, the model assumes that ${L}_{A}>{L}_{Ct}$ and for trials showing guns, ${L}_{A}>{L}_{Cg}$ (Klauer & Voss, 2008).

In contrast, the extended DIM in Fig. 4 assumes that a default racial association is activated automatically, after which a controlled process may or may not intervene. When the controlled process does not intervene, the previously triggered automatic response is given. Therefore, the probability of a fast automatic response (i.e., when the controlled intervention fails) is ${L}_{(1-C)}$. Conversely, the probability of giving a fast response when the controlled process overcomes the prior automatic bias is ${L}_{C}$. Since overcoming the bias takes more time, we assume ${L}_{(1-C)}> {L}_{C}$. Furthermore, the model assumes that overcoming the default automatic bias depends on whether there is a congruence between the image to be identified and the automatically triggered association (e.g., a gun is presented and a bias towards "gun" is triggered automatically) or whether there is an incongruence between the two. As incongruent controlled responses (denoted by ${{L}_{C}}^{-}$) are more costly to overcome than congruent ones (denoted by ${{L}_{C}}^{+}$), the former are assumed to be slower than the latter. Therefore, the DIM model assumes that ${L}_{(1-C)}> {{L}_{C}}^{+}>{{L}_{C}}^{-}$ (Laukenmann et al., 2023).

Regarding parametric modeling of RTs using MPT models, there are essentially two proposals^3^ (Heck, et al., 2018; Klauer & Kellen, 2018). Both involve assuming specific distribution functions for continuous data and estimating the parameters that characterize them. The approach by Klauer and Kellen (2018) assumes a strict serial order of the involved cognitive processes with independent processing times. In contrast, the approach by Heck et al. (2018) does not require the assumption that the processes strictly follow one another, but does allow for modeling individual variability in the parameters. Also, the former approach assumes specific distributions for the latent processes (i.e., sums of a truncated normal and one or more exponential distributions), whereas the latter approach allows for the specification of different distributional families.

In the present study, we focus on the parametric procedure by Heck et al. (2018). In this approach, each branch of the original MPT model (Figs. 1 and 2) is associated with a distribution function characterized by a vector of parameters, denoted as ${\boldsymbol{\eta}}$ (e.g., means and standard deviations of a normal distribution). For the PCRM model, we estimate three vectors of parameters ${{\boldsymbol{\eta}}}_{A}$, ${{\boldsymbol{\eta}}}_{Ct}$, and ${{\boldsymbol{\eta}}}_{Cg}$, which represent the parameters associated with the distributions for automatic processing, controlled processing when a tool is present, and controlled processing when a gun is present, respectively. Conversely, the DIM assumes the parameters ${{\boldsymbol{\eta}}}_{A}$, which represent the parameters characterizing the distributions associated with automatic states, ${{{\boldsymbol{\eta}}}_{C}}^{+}$ for congruent controlled processing, and ${{{\boldsymbol{\eta}}}_{C}}^{-}$ for incongruent controlled processing. Given that all ${\boldsymbol{\eta}}$ parameters correspond to a certain distributional family, the predictions made by each model (e.g., that responses derived from automatic processes are faster than controlled ones) will depend on how RTs are assumed to be distributed. Overall, the modeling assumptions for RTs depend both on the assumed probability density function (PDF) and the set of equality constraints on the RT parameters.

Once RTs are included in an MPT model, we can test the validity of RT distributional assumptions of each model or compare models that differ in their RT predictions. By incorporating RTs into the analysis, we can assess whether the observed data more closely match the predictions of the DIM or PCRM, thereby testing the validity of the models. As mentioned above, it is only possible to test the two models against each other when extending the corresponding MPT models with RTs. However, it is currently unclear whether parametric or non-parametric approaches are better suited for this. If the models are in line with the data-generating process, the parametric procedure will have higher statistical power than the nonparametric method (Heck et al., 2018; Heck & Erdfelder, 2016). Currently, however, we do not know by how much statistical power decreases when relying on the nonparametric model and which factors may moderate this loss in power. Moreover, the performance of parametric models may be very sensitive to discrepancies between the actual distribution of the data and the distributional assumptions of the fitted model (Klauer & Kellen, 2018). To the best of our knowledge, no study has systematically compared the two modeling approaches by Heck and Erdfelder (2016) and Heck et al. (2018) and identified factors that may benefit one approach over the other.

The present study thus aims to compare the performance of parametric and nonparametric approaches for RT-extended MPT models. We investigate conditions in which either the parametric or the nonparametric approach is expected not to perform well. To do so, we conducted a Monte Carlo simulation study using the PCRM and the DIM, the two models for the weapon identification task outlined above (Payne, 2001; Rivers, 2017). The article includes three simulation studies that address different goals.

In Simulation 1, we assess the calibration and statistical power of the goodness-of-fit test of the parametric modeling approach (Heck et al., 2018), which rests on a non-standard test statistic, namely, the Dzhaparidze–Nikulin statistic (Dzhaparidze & Nikulin, 1975). We also study the loss in power of the goodness-of-fit test when relying on the nonparametric approach. With regard to the parametric approach, we also examine whether certain types of misspecified distributional assumptions can still show an acceptable fit to simulated data. Only models with acceptable goodness-of-fit will be considered in subsequent simulations. Additionally, in this section, we also evaluate the implementation of the Dzhaparidze–Nikulin statistic and its performance in fitting small sample sizes.

In Simulation 2, the competing modeling approaches will be compared in terms of statistical power to test the RT predictions of each model. The likelihood ratio test (LRT) will be applied to compare nested models that make different assumptions about discrepancies in the RT distributions between different branches. Studying the properties of nested model tests is important to enable future validation studies that focus on core assumptions made by the PCRM and DIM about the ordering of latent RT distributions (e.g., the DIM assumes that automatic responses are faster than congruent controlled responses, and these are faster than incongruent controlled responses).

Finally, in Simulation 3, we will evaluate the sensitivity of the modeling approaches for selecting between competing non-nested models. It is only possible to test the DIM and the PCRM against each other if we extend the corresponding MPT models with RTs. However, this raises the question of the suitability of the approaches for selecting between the two models. Therefore, we will investigate the factors affecting the ability to recover the true model using model weights based on the Akaike information criterion (wAIC; Burnham & Anderson, 2004). Studying the performance of RT-extended MPT models in terms of model recovery is relevant for the choice of interventions aimed at reducing racial biases (Calanchini et al., 2021; Gonsalkorale et al., 2011).

## Simulation 1

In Simulation 1, we study the power of goodness-of-fit tests for parametric and non-parametric models. Specifically, we investigate whether the goodness-of-fit test based on the Dzhaparidze–Nikulin statistic (Dzhaparidze & Nikulin 1975), a generalized χ^2^ statistic introduced for parametric MPT-RT models by Heck et al. (2018), adheres to the expected nominal type I error level. This is done to check its implementation in the *gpt* package in R by Heck et al. (2018), since previous validations focused only on a much simpler MPT model, and to assess the performance of the test in small samples. Only if the test is well calibrated can we use it to detect which models fit the data adequately and which do not in the next step.

For the first time, we also systematically assess the extent of the statistical loss in power when relying on the more robust, nonparametric approach by Heck and Erdfelder (2016) instead. With respect to distributional assumptions of parametric models, we will also study which distributional families and constraints are useful for capturing the RT data simulated from a specific distributional family (e.g., the shifted Wald distribution). The performance of the models will be evaluated in situations where we manipulate the number of observations, *N*, the effect size of the differences between the RT distributions of different branches, and the nature of these differences in terms of location, standard deviation, or shape. Models that show a low rejection rate of the goodness-of-fit test will be further investigated in Simulations 2 and 3.

### Simulation conditions

For all simulation conditions, 2000 data sets were generated based on the PCRM or the DIM with a number of trials per tree varying on different levels, $n=100$ or $n=300$. The total number of observations $N$ corresponds to the sum of the frequencies for the racial priming of black and white faces and the images of tools and guns (four trees: black-tool, black-gun, white-tool, white-gun). Hence, each simulation condition followed a balanced factorial design with $N=4\cdot n.$ The simulation conditions for the sample size were chosen based on the experimental studies included in the MPT-RT reanalysis by Laukenmann et al. (2023), in which $N$ ranged between 200 (as in Madurski & LeBel 2015) and 1100 (as in Amon & Holden 2016).

The probability parameters of automatic processes (see Figs. 1 and 2) were set to ${A}_{b}=.80$, ${A}_{w}=.20$ ${C}_{b}=.70$ and ${C}_{w}=.65$. These values were determined by taking into account the range of parameter estimates reported in the reanalyses by Laukenmann et al. (2023). Furthermore, the parameters were set to ensure an adequate number of observations of controlled responses and discrepancies between automatic and controlled processes, thus enabling the distinguishability and identifiability of the PCRM and DIM.

Regarding the continuous distributions, simulated RT data followed a shifted Wald distribution, which is characterized by the parameters $\mu$ (location/scale), $\lambda$ (shape), and $\delta$ (shift). Table 1 shows the chosen parameter values ${\boldsymbol{\eta}}$ as a function of the manipulation of the effect size of the inter-branch differences and the nature of these differences ($\Delta \mu$ and $\Delta \lambda$, scale and shape simulation conditions) for the PCRM and DIM model.

**Table 1:** Parameters **η** of the shifted-Wald distribution used for simulating RT data

Effect size		PCRM							DIM		
		Free* η*			Fixed* η*		Free* η*			Fixed* η*	
		${\lambda }_{A}$	${\lambda }_{Ct}={\lambda }_{A}+\Delta \lambda$	${\lambda }_{Cg}={\lambda }_{A}+2\Delta \lambda$	$\mu$	$\delta$	${\lambda }_{\left(1-C\right)}$	${\lambda }_{C+}={\lambda }_{(1-C)}+\Delta \lambda$	${\lambda }_{C-}={\lambda }_{(1-C)}+2\Delta \lambda$	$\mu$	$\delta$
$\Delta \lambda$	0	150	150	150	100	200	150	150	150	100	200
	100	150	250	350	100	200	150	250	350	100	200
	200	150	350	550	100	200	150	350	550	100	200
	300	150	450	750	100	200	150	450	750	100	200
		${\mu }_{A}$	${\mu }_{Ct}={\mu }_{A}+\Delta \mu$	${\mu }_{Cg}={\mu }_{A}+3\Delta \mu$	*λ*	$\delta$	${\mu }_{\left(1-C\right)}$	${\mu }_{C+}={\mu }_{(1-C)}+\Delta \mu$	${\mu }_{C-}={\mu }_{(1-C)}+3\Delta \mu$	*λ*	$\delta$
$\Delta \mu$	0	100	100	100	150	200	100	100	100	150	200
	25	100	125	175	150	200	100	125	175	150	200
	50	100	150	250	150	200	100	150	250	150	200
	75	100	175	325	150	200	100	175	325	150	200

The ${\boldsymbol{\eta}}$ parameters for data generation correspond either to the PCRM or the DIM (*left* and *right columns*, respectively). For each model, we distinguish between the parameters “Free ${\boldsymbol{\eta}}$*”* (which vary across branches) and “Fixed ${\boldsymbol{\eta}}$*”* (which are constant across branches). The rows represent different effect sizes in terms of differences between branches (induced by manipulating $\lambda$ and $\mu$, respectively). For the PCRM, the values of *∆λ* (*top rows*) and *∆* $\mu$ (*bottom rows*) have the following subscripts: $A=$ automatic processing; $Ct=$ controlled processing for tools; and $Cg=$ controlled processing for guns. The parameters for the DIM (*right column*) include the following subscripts: $\left(1-C\right)=$ automatic processing (no control); ${C}^{+}=$ congruent controlled processing; and ${C}^{-}=$ incongruent controlled processing

As shown in Table 1, in all simulation conditions, the shift parameter $\delta$ was set to 200 ms to represent non-decision time. Therefore, the model does not allow for the possibility of generating RTs with a shorter time than required to process information and make a decision. In the $\Delta \lambda$ conditions (top four rows), discrepancies between latent RT distribution components were generated by manipulating the shape parameter $\lambda$ while fixing the scale parameter $\mu$ to $100$. In contrast, in the $\Delta \mu$ conditions (bottom four rows), we manipulated the $\mu$ parameter to induce discrepancies between distinct distributional components while holding $\lambda$ constant at $150.$ For the $\Delta \mu$ conditions, we assumed effect sizes of $\Delta \mu$ = {0, 25, 50, 7} and for the $\Delta \lambda$ conditions, we assumed effect sizes of $\Delta \lambda$ = {0, 100, 200, 300}.

Discrepancies between latent RT distributions of different MPT branches were generated relative to the fastest branch in each model. The fastest processes are the automatic processing branch (subscript $A$) in the PCRM and the failed controlled intervention branch (subscript $1-C$) in the DIM. The parameters of the second fastest state (with subscript $Ct$ for controlled processing for tools in the PCRM; and subscript $C$+ for congruent controlled processing in the DIM) are set by adding $\Delta \lambda$ or $\Delta \mu$ to the $\lambda$ or $\mu$ parameters that characterize the fastest branch, respectively. The RT distribution of the slowest states (with subscript $Cg$ for controlled processing of guns in the PCRM; and $C$- for incongruent controlled processing in the DIM) were manipulated by adding $2\cdot \Delta \lambda$ or $3\cdot \Delta \mu$. These specifications can also be seen in the formulas included in the subheadings of Table 1 and were established to generate RTs that are ordered in terms of relative processing speed.

**Fig. 5:**
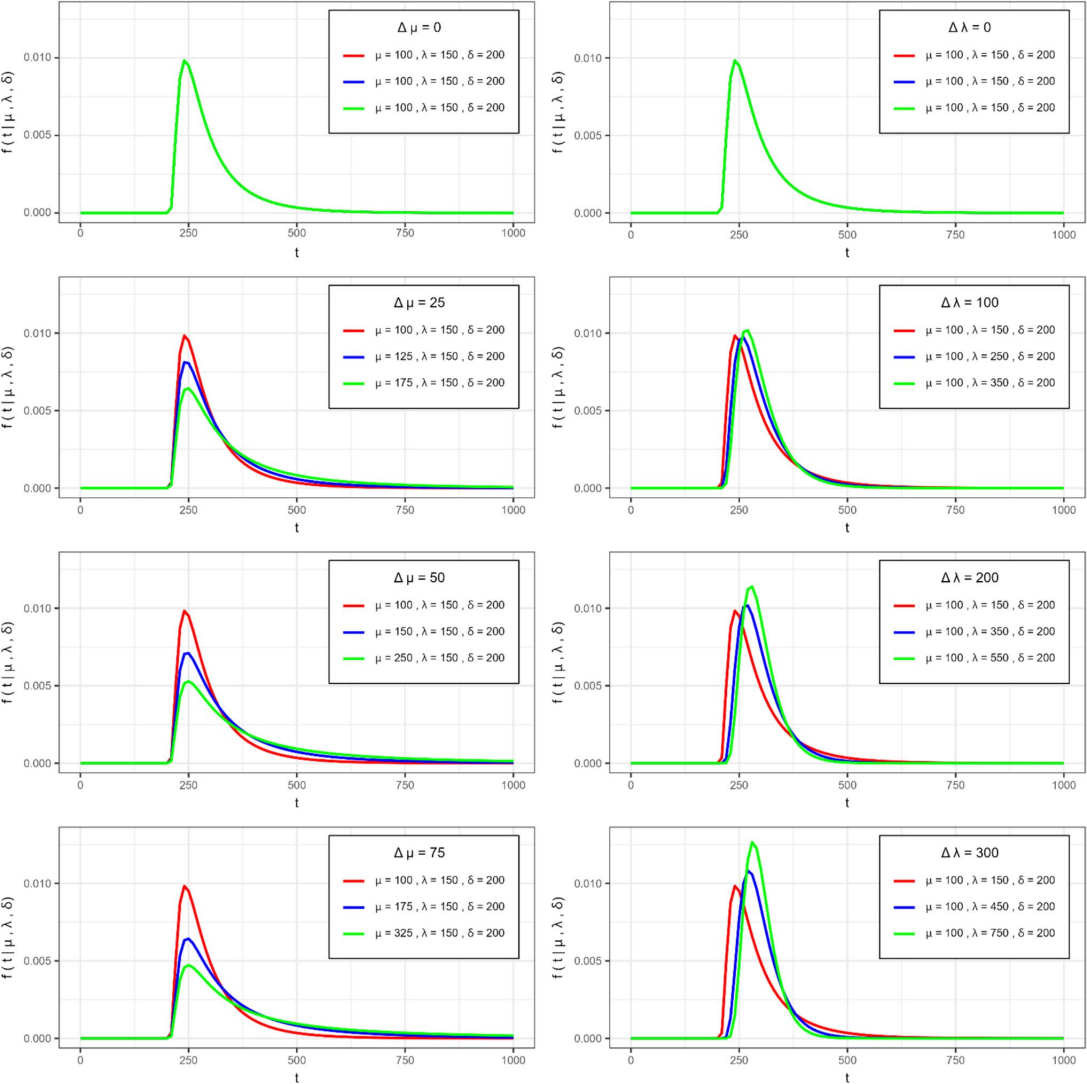
Probability density functions (PDFs) of the three shifted-Wald distributions of RTs used for data generation. *Note*. The left column shows the PDFs of RTs simulated in the *∆µ* simulation conditions for the effect sizes ∆µ = {0, 25, 50, 75} (top to bottom rows). The right column shows the *∆*$\lambda$ simulation conditions for the effect sizes *∆*$\lambda$ = {0, 100, 200, 300} (top to bottom rows). The three colors correspond to the distributions associated with the fastest (*red*), medium (*blue*), and the slowest (***green***) branch of each model

Figure 5 illustrates the PDFs of RTs corresponding to the three distinguishable MPT model branches (fastest in red, medium in blue, and slowest in green) for each simulation condition. Each row shows increasing effect sizes, with greater differentiation among the three branches, either by manipulating the $\mu$ parameter (left column) or the $\lambda$ parameter (right column). In the shifted Wald distribution, $\mu$ determines the scale of the PDFs without significantly shifting the peak. Increasing $\Delta \mu$ (from top to bottom in the left column) broadens the distribution and increases the expected mean. Conversely, $\lambda$ affects the distribution's shape, tightening it around the mean and shifting the peak location, with increased $\lambda$ (from top to bottom in the right column) resulting in reduced variance without altering the mean.

Note that Fig. 5 does not differentiate between the DIM and PCRM models, as the effect sizes and parameter values are the same for both models. What differs between the two models is which branch is classified as faster, medium, or slower. All datasets generated for Simulations 1, 2, and 3 are available at the Open Science Framework (https://osf.io/54mdq/).

### Fitting conditions

For the simulation conditions where the PCRM was used to generate the data, we fitted both parametric and non-parametric PCRM models. Similarly, for the data generated by the DIM, we fitted both parametric and non-parametric DIM versions. Regarding the parametric approach, we fitted models with different assumptions about the distribution of RTs. The assumptions are contingent upon two factors:

1. the chosen latent RT probability density function, which includes the shifted Wald, ex-Gaussian, shifted lognormal, shifted gamma, and normal distribution; and
2. the equality constraints that determine how RTs are assumed to differ across branches.

To ensure comparability across parametric models, we introduced three RT component distributions (fast, medium, and slow) that correspond to distinct patterns of parameter constraints across MPT branches. Similar to the data-generating conditions for the Wald distribution, the simulation compared three conditions for fitting the models with different parameter constraints. In condition R1, both $\mu$ and *λ* were allowed to vary freely without equality constraints across component distributions. In R2, *λ* was assumed to be identical for all branches, while no restrictions were set on the parameter $\mu$, allowing it to vary freely. In R3, the parameter *λ* was allowed to vary while keeping $\mu$ fixed. Technically, we captured the effects of the three distributional components (fast, medium, and slow) in model fitting by defining and estimating suitable additive shift parameters (termed $d$ and $s$), which act as incremental modifications to the original parameters of the RT distributions.

The models fitted with distributions other than the data-generating shifted Wald also require a vector ${\boldsymbol{\eta}}$ with parameters characterizing the PDF in terms of mean, variance, location, scale, or shape. Pattern of constraints on ${\boldsymbol{\eta}}$ were applied similarly to those used for fitting the shifted Wald under R1, R2, and R3. These constraints were visually derived (see Appendix B) by considering how the shifted Wald parameters influence the shape of RT distributions. Table 2 provides an overview of how this parametrization was applied for all distributions.

**Table 2:** Parameterization of RT component distributions used for model fitting in the conditions R1, R2, and R3

Distribution	Parameters	Fast branch	Medium branch	Slow branch
Shifted Wald	location/scale, shape, shift	$\mu$*, * $\lambda$*,* $\delta$	$\mu + {d}_{1}, \lambda + {s}_{1}, \delta$	$\mu + {d}_{1} + {d}_{2}, \lambda + {s}_{1} + {s}_{2}, \delta$
Ex-Gaussian	tau, SD, shift	$\tau , \sigma , \mu$	$\tau + {d}_{1}, \sigma + {s}_{1}, \mu$	$\tau + {d}_{1}+ {d}_{2}, \sigma + {s}_{1} + {s}_{2}, \mu$
Shifted lognormal	location/scale, SD, shift	$\mu , \sigma , \delta$	$\mu +{d}_{1} , \sigma + {s}_{1}, \delta$	$\mu + {d}_{1} + {d}_{2}, \sigma + {s}_{1}+ {s}_{2}, \delta$
Normal	mean, SD	$\mu , \sigma$	$\mu + {d}_{1} , \sigma + {s}_{1}$	$\mu + {d}_{1} + {d}_{2}, \sigma + {s}_{1}+ {s}_{2}$
Shifted gamma	scale, shape, shift	$\theta , k, \delta$	$\theta +{d}_{1} , k + {s}_{1}, \delta$	$\theta + {d}_{1} + {d}_{1} , k + {s}_{1} + {s}_{2}, \delta$

In R1, all parameters in Table 2 are estimated, allowing all additive *d* and *s* parameters to vary freely. In R2, we impose constraints to ensure that only the parameters approximating the effects of increasing the location/scale $\mu$ in the Wald distribution are allowed to vary. This implies that *s₁ = s₂ = 0* for all RT distributions (see Appendix B, Figure B1). Following this logic, R3 allows fitting parameters that produce effects approximately similar to those generated in the Wald distribution when only increasing $\lambda$, thereby restricting *d₁ = d₂ = 0* to achieve comparable distributional changes (see Appendix B, Figure B2). In all cases, the shift parameter of each distribution, usually assumed to reflect non-decision times, remained fixed (see Appendix B, Figure B3). All parameters were restricted to be estimated within the range of 0.001 to 1000, thereby ensuring that the model incorporates the order constraint RT_fast_ < RT_medium_ < RT_slow_ across component distributions.

The above parametrization for model fitting does not ensure a perfect equivalence of parameter constraints or RT distributions between fitting conditions when assuming different distributional families. Nevertheless, this approximation allows us to investigate whether RT-extended MPT models with misspecified distributional assumptions (but comparable sets of constraints) can be used to mimic and recover certain patterns of latent RT distributions generated by the shifted Wald model.

The DIM and the PCRM can only be distinguished empirically because they assume different constraints on latent RT distributions, which in turn result in different predictions for observed RT distributions. The non-parametric approach makes the following assumptions on the latency parameters $L$, which quantify the relative speed of different latent processes. Note that the constraints are in line with the data-generating mechanism:

- PCRM: $${L}_{A}>{L}_{Ct}>{L}_{Cg}$$
- DIM: $${L}_{A}>{L}_{C+}>{L}_{C-}$$

Similarly, the parametric approach makes the following constraints on the parameters of the shifted Wald distribution:

- PCRM: ${\mu }_{A}<{\mu}_{Ct}<{\mu}_{Cg}$ and/or ${\lambda}_{A}<{\lambda }_{Ct}<{\lambda}_{Cg}$ (depending on the data-generating condition)
- DIM: ${\mu }_{A}<{\mu }_{C+}<{\mu }_{C-}$ and/or ${\lambda }_{A}<{\lambda }_{C+}<{\lambda }_{C-}$

As for the R packages used for model fitting, we relied on the *MPTinR* package (Singmann & Kellen, 2013) for non-parametric models and the *gpt* package (Heck et al., 2018) for parametric models.

### Performance measures

To assess the performance of the non-parametric and parametric approaches in terms of goodness-of-fit, we investigated the rejection rates of the goodness-of-fit test via Monte Carlo simulations. Goodness-of-fit tests are provided by the *MPTinR* package for the non-parametric approach (Singmann & Kellen, 2013) and by the *gpt* package for the parametric approach (Heck et al., 2018). The former uses the LRT statistic ${G}^{2}$ while the latter uses the Dzhaparidze–Nikulin test statistic (Dzhaparidze & Nikulin, 1975). In both cases, the null hypothesis assumes that there is no difference between the predicted and the observed data. The null hypothesis is rejected if the *p* value is smaller than the standard significance level of $\alpha$ = 5%. Using Monte Carlo simulations, the rejection rate (RRate) can be estimated using the relative frequency of significant test results:

1 $$\widehat{RRate}=\frac{1}{M}{\sum }_{i=1}^{\mathrm{M}}1\left({p}_{i} < .05\right)$$

The RRate reflects either the type I error rate when the true effect is zero or the statistical power when the true effect is different from zero. It is obtained by counting the number of iterations in which the *p* value is smaller than.05—using the indicator function—and dividing by the total number of simulations, *M*.

### Results

**Fig. 6:**
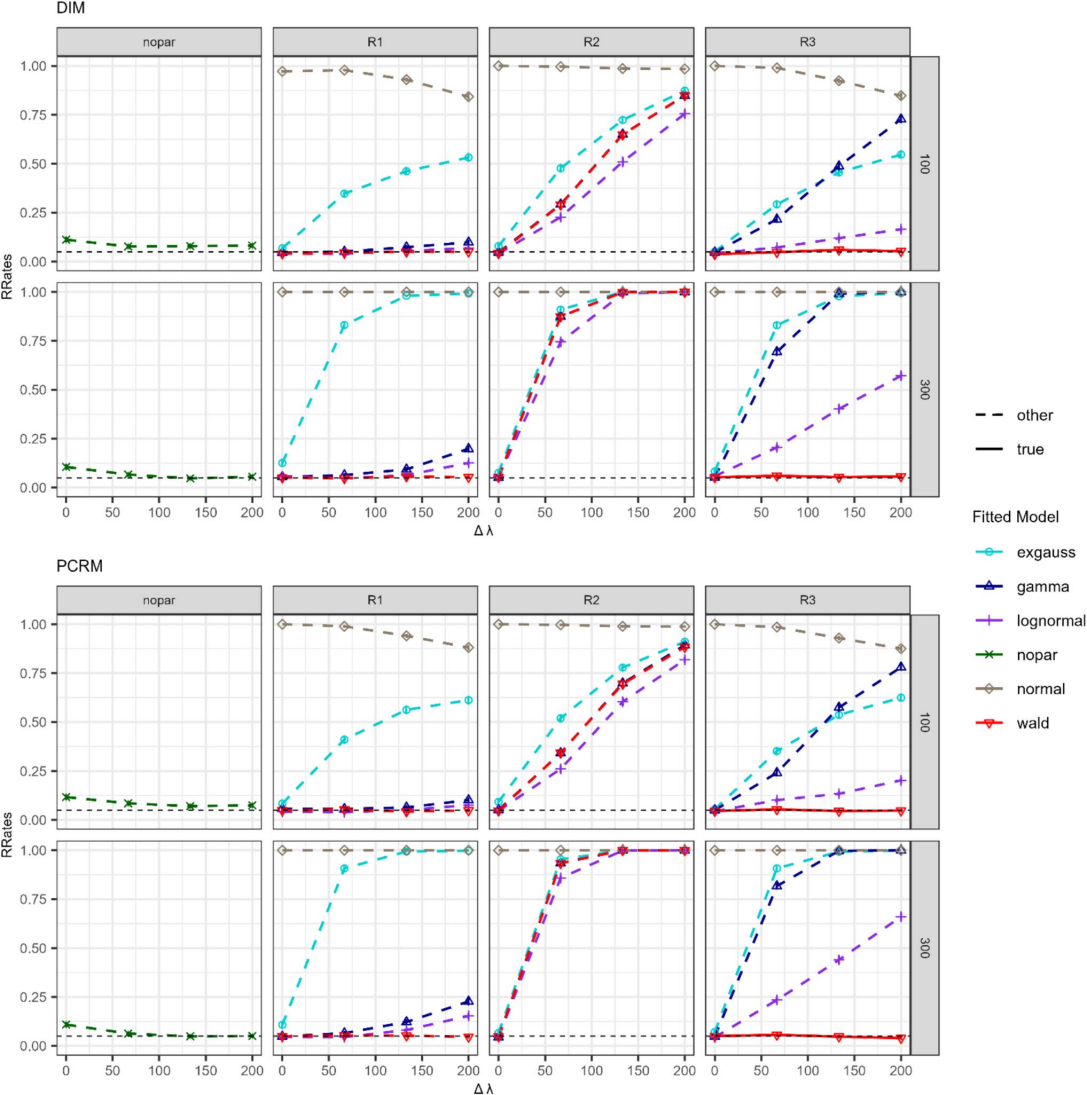
Rejection rates (RRates) for the goodness-of-fit test for the ${\Delta\lambda}$ manipulation conditions in Simulation 1. *Note.* Effect sizes were manipulated using *∆*$\lambda$ values ranging from 0 to 300. DIM (*top two rows*) and PCRM (*bottom two rows*) refer to the data generation and fitting processes, respectively. The row title denotes the number of observations per tree (100 or 300) and the column one the type of constraint (R1, R2, and R3). For the Wald distribution, R1 allows both the location/scale and the shape parameters to vary freely across branches; R2 only allows the location/scale parameters to vary freely; and R3 only allows the shape parameters to vary freely (for the other distributions, see Table 2). Fitted models are distinguished by colors and shapes. The correctly specified fitted model (used to generate the data) is represented by a *solid line*, while all other models are shown by *dashed lines*

**Fig. 7:**
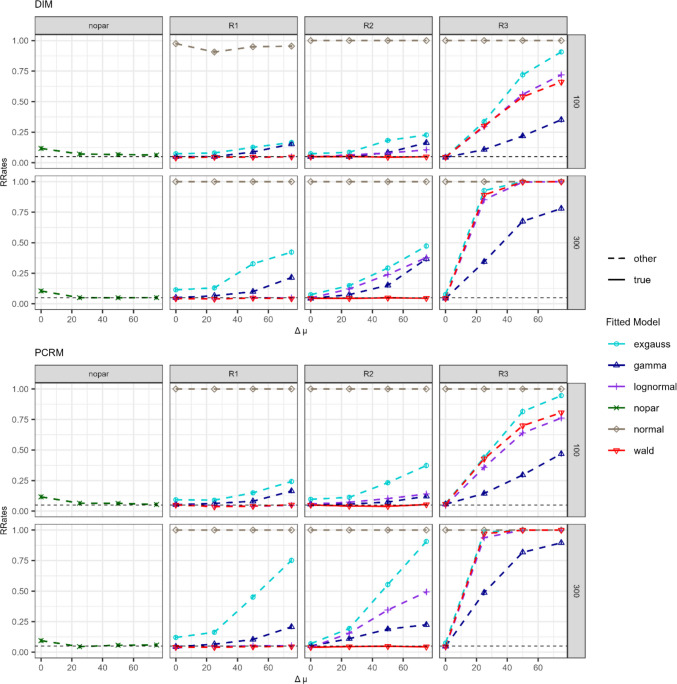
Rejection rates (RRates) for the goodness-of-fit test for the ${{\Delta} {\mu}}$ manipulation conditions in Simulation 1. *Note*: RT differences were manipulated using *∆µ* values ranging from 0 to 75. DIM (*top two rows*) and PCRM (*bottom two rows*) refer to the data generation and fitting processes, respectively. Each row title indicates the number of observations per tree (100 or 300), while each column title shows the type of constraint (for the other distributions, see Table 2). For the Wald distribution, R1 allows both $\mu$ and $\lambda$ to vary across branches, R2 allows only $\mu$ to vary, and R3 allows only $\lambda$ to vary. Fitted models are distinguished by color and shape. The correctly specified fitted model (used to generate the data) is represented by a *solid line*, while all other models are shown by *dashed lines*

Figures 6 and 7 show the RRates of the goodness-of-fit test for the $\Delta \lambda$ and $\Delta \mu$ simulation conditions, respectively. On the *y*-axis, RRates are plotted for several levels of effect size (*x*-axis). The *upper two rows* correspond to conditions where the DIM was used for simulating and fitting data, whereas the *bottom two rows* correspond to the PCRM. Plot titles show the number of observations per branch, with $n=100$ in the first and third rows, and $n=300$ in the second and fourth rows. To evaluate whether the Dzhaparidze–Nikulin statistic was correctly implemented and is unaffected by low sample sizes, we analyzed whether, whenever the fitted model matched the data-generating one, RRates adhered to the expected nominal significance level of α = 5% (depicted as red solid lines in Figs. 6 and 7). In the $\Delta \lambda$ conditions (see Fig. 6), the correctly specified Wald model with free $\lambda$ parameter across branches (solid line; R3 constraints in the right column) adhered to the expected type I error level. Similarly, for the $\Delta \mu$ conditions in Fig. 7, fitting the true model with R2 constraints (i.e., a shifted Wald model allowing $\mu$ to vary freely) resulted in RRates close to the nominal significance level of 5%. Furthermore, these results were consistent regardless of the number of observations or the type of model (DIM or PCRM). The Dzhaparidze–Nikulin statistic implemented in the *gpt* package (Heck et al., 2018) maintained the nominal type I error rate for the goodness-of-fit test, with no substantial violations observed for the simulated sample sizes.

In the following, we consider the results for the goodness-of-fit test when using the nonparametric instead of the parametric approach. Figures 6 and 7 show that there were no relevant differences between RRates when fitting the correctly specified parametric model (solid red line) or the corresponding non-parametric model (green line in the left column). Hence, the goodness-of-fit test for the nonparametric approach mostly adhered to the nominal significance level, similar to the parametric model.

Next, we investigated whether parametric models with misspecified distributional assumptions could mimic the data-generating distributions. Most of the fitted parametric models assumed distribution families suitable for the right skewness of RTs (with the exception of the normal distribution). These models fitted the data well when the true RT distributions were identical for all branches (i.e., $\Delta\lambda$ = 0 or $\Delta\mu$ = 0). However, RRates increased for larger effect sizes. Specifically, Fig. 6 shows that, under the $\Delta\lambda$ manipulation and when fitting the R2 and R3 constraints (where only one free parameter could vary across latent RT distributions), RRates for all models exceeded the significance threshold *α* when effect sizes were non-zero. This pattern was particularly evident for larger sample sizes (e.g., $n=300$). Nevertheless, it is worth noting that the lognormal model consistently exhibited comparatively lower recovery rates (RRates) relative to the other misspecified parametric models. Under the fitting condition R1, where two parameters could vary across latent RT distributions (i.e., only the non-decision time parameter was assumed to be identical for all MPT model branches), the gamma distribution (blue line) and the lognormal distribution (purple line) could mimic the simulated data, as indicated by low RRates. In none of the conditions did the normal model (light brown line) mimic the simulated left-skewed RT distributions. The ex-Gaussian model (light blue line) did not fit the data when effect sizes differed from zero.

Concerning the $\Delta\mu$ simulation conditions in Fig. 7, when fitting parametric models with misspecified RT distributions, the best fit was achieved when allowing two parameters to vary across branches (R1 constraints). However, it is worth noting that under R2 conditions, where only one free parameter was permitted to mimic the effects of manipulating the Wald parameter $\mu$, RRates were not substantially lower than those observed under R1. Hence, both gamma and lognormal could be feasible parametric alternatives for capturing differences in $\mu$ of the Wald distribution under R1 and R2 constraints. As in previous analyses (Fig. 6), the normal distribution generally failed to fit data generated by the Wald distribution. The ex-Gaussian model also performed poorly under the conditions involving larger effect sizes on the tested RT differences and larger sample sizes. Nonetheless, the ex-Gaussian could account for effects corresponding to the Wald parameter $\mu$ more accurately than those related to the parameter $\lambda$.

### Discussion

Simulation 1 shows that the Dzhaparidze–Nikulin goodness-of-fit test, proposed for parametric MPT-RT models by Heck et al. (2018), adhered to the nominal type I error level, with no violations observed even in small sample sizes. Additionally, the standard goodness-of-fit test for the non-parametric approach by Heck and Erdfelder (2016) was very robust as indicated by rejection rates close to 5% in all simulation conditions, regardless of the model version (PCRM or DIM), the number of observations, or the type of manipulation of RT differences ($\Delta\mu$ and $\Delta\lambda$ manipulation conditions).

Regarding the analysis of parametric models with misspecified distributional assumptions, parametric models (Heck et al., 2018) were quite sensitive to discrepancies between simulated data and distributional assumptions. Since a key characteristic of the parametric approach is that it evaluates the adequacy of data to a very specific distribution, it is a benefit that deviations from this distribution are detected with considerable power, even in cases with small sample sizes. As expected, this effect was particularly pronounced when the number of observations or effect sizes became larger, as the power to detect discrepancies between observed and fitted aspects of the data increased. This highlights the importance of using the parametric approach only when we have strong assumptions about the distribution of the data. The distributional families that performed best at capturing RT distributions generated by shifted Wald distributions were the shifted lognormal and shifted gamma models. Only the set of constraints in the fitting condition R1 proved to be capable of adapting to the manipulations applied to the shifted Wald, which corresponded to changes both in the expected mean, variance, and shape of the RT component distributions. This set of constraints allowed two free parameters for each latent RT distribution and only fixed the non-decision time parameter to be identical across all MPT model branches. Thus, in Simulations 2 and 3, we fitted only parametric models assuming shifted Wald, lognormal, or shifted gamma distributions with two free parameters per latent state (R1 constraints), as well as the nonparametric model.

## Simulation 2

Parametric and nonparametric procedures differ in how they incorporate RTs, and we do not know to what extent either can effectively detect discrepancies in RT distributions across branches. As a remedy, we performed a simulation study using the likelihood ratio test (LRT) to select between unrestricted and nested versions of the DIM and the PCRM.

Fitting the data-generating (and thus, the correctly specified) parametric model should be optimal in terms of the statistical power for detecting discrepancies in RT distributions. In comparison, fitting a non-parametric model is expected to result in a loss of power. However, neither method has yet been systematically compared. Hence, it is difficult to determine to what extent statistical power decreases when using a nonparametric (Heck & Erdfelder, 2016) instead of a parametric approach (Heck et al., 2018) and which factors affect this loss. As a remedy, Simulation 2 aims to compare the performance of different RT-extended MPT models for testing the ordering of latent processes assumed by the DIM and the PCRM. We evaluated the performance of different model versions under conditions expected to impact the power of the test, including different sample sizes *N*, different effect sizes of RT discrepancies across branches, and different assumptions about the nature of these discrepancies (e.g., location/scale changes or shape changes).

Furthermore, the performance of the parametric procedure is expected to depend on whether assumptions about the underlying distributional families are correctly specified. Therefore, we will also evaluate parametric models that assume other distributional families. However, to assess the robustness of the parametric procedure to misspecification, we focus only on model variants that have already shown acceptable goodness-of-fit in Simulation 1.

### Simulation conditions

The same data-generating conditions as in Simulation 1 were implemented.

### Fitting conditions

In Simulation 2, we fitted the same model versions outlined in Simulation 1, but included only the shifted Wald, gamma, and lognormal distributions with R1 constraints (i.e., only the shift parameter was fixed across branches, while the other two parameters were free). In addition, a second, nested version of each model (Model_eq_) was fitted assuming that there are no RT discrepancies across the MPT model branches. This can be implemented for the non-parametric approach by restricting the parameters such that all latency parameters $L$ are equal,

- PCRM: $${L}_{A}={L}_{Ct}={L}_{Cg}$$
- DIM: $${L}_{A}={L}_{C+}={L}_{C-}$$

while in the parametric approach^4^,

- PCRM: $${\mu }_{A}={\mu }_{Ct}={\mu }_{Cg}$$ and $${\lambda}_{A}={\lambda}_{Ct}={\lambda}_{Cg}$$
- DIM*:* $${\mu}_{A}={\mu}_{C+}={\mu}_{C-}$$ and $${\lambda}_{A}={\lambda}_{C+}={\lambda}_{C-}$$

### Performance measures

To compare the power of the approaches for evaluating RT discrepancies across branches, we conducted nested model comparisons for both the PCRM and the DIM. For the PCRM, we tested the null hypothesis that there are no differences in RTs among automatic, gun-controlled, and tool-controlled states. Similarly, for the DIM, we examined the absence of RT differences among automatic, congruent-controlled, and incongruent-controlled states. This involved testing models assuming three distinct RT distributions (subscript “*full”*) against models that assume a single, shared RT distribution across all branches (subscript “*eq*,” indicating equal RT distributions). The LRT relied on the standard ${G}^{2}$ statistic for nested models. Based on the LRT, we calculated the RRate (see Eq. 2) of rejecting the null hypothesis, that is, the relative frequency of replications in which Model_eq_ resulted in a significantly worse fit than Model_full_.

### Results

**Fig. 8:**
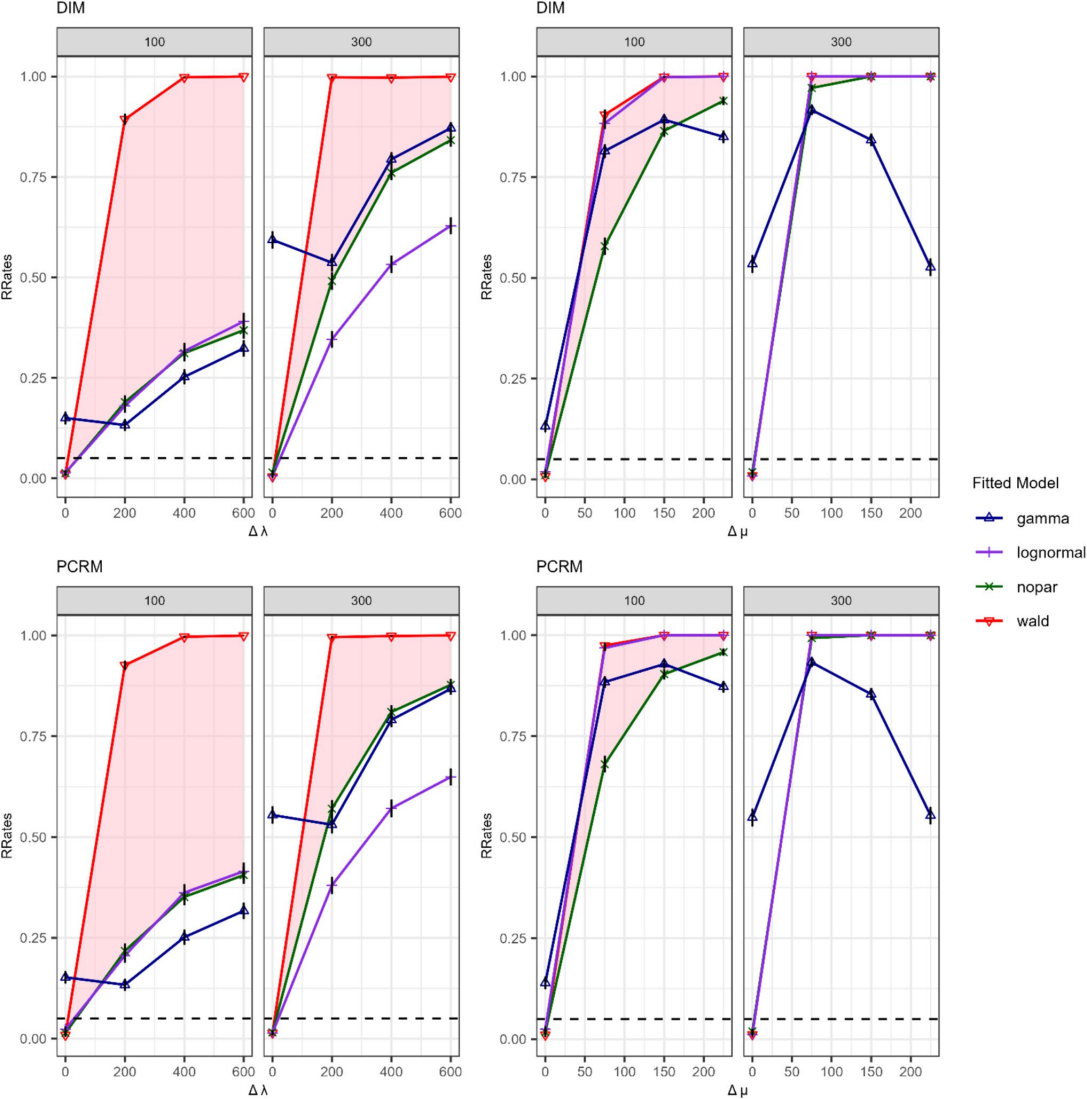
Rejection rates (RRates) of the nested model test in Simulation 2. *Note.* Differences in shifted Wald distributions were manipulated via the *∆µ* parameter (*two right columns*) and the *∆*$\lambda$ parameter (*two left columns*). DIM (*top row*) and PCRM (*bottom row*) refer to the data generation and fitting processes. The title of each plot denotes the number of observations per tree (100 or 300). The *light-red area* highlights the loss in power when relying on the nonparametric model rather than the correctly specified parametric model.

Figure 8 illustrates RRates for nested model comparisons for varying levels of the effect sizes $\Delta\lambda$ and $\Delta\mu$. The left column in Fig. 8 illustrates the effect of manipulating the shape parameter $\Delta\lambda$. The correctly specified, shifted Wald model adhered to the nominal $\alpha$ level when no effects were present, while showing high power for detecting differences in RT distributions even for small effect sizes. The nonparametric model also adhered to the expected RRate of 5% $5\%$when no effects were present, while still showing high RRates for large sample and effect sizes, it displayed significantly lower power than the correctly specified parametric model in all other conditions (highlighted by red areas). Indeed, when the sample size was small ($n=100$), the non-parametric approach exhibited a loss of power to detect shape discrepancies across both small and large effect sizes (between 70.5% in $\Delta\lambda=200$ and 64% $\Delta\lambda=600$). The lognormal model also showed a considerable loss of power relative to the true model under the $\Delta \lambda$ condition, showing an even higher loss of power than the non-parametric model. The gamma model also performed poorly, with RRates substantially exceeding the expected 5% under conditions in which no true RT differences were simulated. In other $\Delta\lambda$ conditions, its power performance was similar to the nonparametric model.

The right column in Fig. 8 illustrates the effect of manipulating the shape parameter $\Delta\mu$. Without a manipulation of the location/scale parameter ($\Delta\mu =0$), the RRate for the data-generating, shifted-Wald model adhered to the nominal significance level of *α* = 5%. As the effect size $\Delta\mu$ increased, RRates for the Wald model (red line) increased and were above 80% across all conditions, indicating high statistical power. The increasing trend also emerged for the non-parametric model (green line), with only a slight loss of power for large sample sizes ($n=300$). With small sample sizes ($n=100$), the non-parametric model (green line) exhibited a loss of power between 33% in ${{\Delta} {\mu}}=75$ and 6% in $\Delta\mu=225$ as indicated by the light red area. The lognormal model (purple line) performed remarkably well under all ${{\Delta} {\mu}}$ conditions showing barely any deviation from true model performance. In contrast, the gamma model (blue line) showed an inflated type I error rate and a pronounced loss of power relative to the Wald model, particularly under large effect sizes. This decline in performance may be attributed to the gamma model’s limited capacity to capture substantial discrepancies in ${{\Delta} {\mu}}$ within the generated distributions. These findings are consistent with those from Simulation 1 (Fig. 7), where the gamma model’s goodness of fit deteriorated markedly at ${{\Delta} {\mu}}$ increased.

### Discussion

The results of Simulation 2 underscore the importance of selecting appropriate models for testing the core assumptions of the PCRM and DIM regarding the ordering of latent processes. Statistical power to detect RT differences varied depending on whether the manipulation involved the location/scale parameter (${{\Delta} {\mu}}$)—which produces more pronounced distributional shifts—or the shape $(\Delta\lambda$), which yields subtler distributional changes (see simulated changes in Fig. 5).

While it is expected that the non-parametric model exhibits a loss of power compared to the true parametric model, it is informative that this loss can be relatively small under certain conditions – specifically, when sample sizes are large and when the discrepancies affect the location/scale of RT distributions. In contrast, its performance declined substantially for small sample sizes and when differences in RT distributions were only subtle, such as the ones induced by varying the shape parameter $\lambda$. Aside from this limitation, the non-parametric model remains a valuable tool due to its flexibility and robustness in the absence of strong parametric assumptions. It often outperformed incorrectly specified parametric alternatives, including parametric MPT models with standard choices such as the lognormal or gamma.

Regarding the robustness of parametric models other than the data-generating model, the lognormal model performed poorly when accounting for changes induced by the $\Delta\lambda$ parameter of the shifted-Wald distribution, exhibiting greater power loss than the non-parametric model. In contrast, it showed excellent performance for detecting changes driven by $\Delta \mu$, outperforming the non-parametric model, especially in small sample sizes. In general, however, the gamma model failed to mimic manipulations of the shifted-Wald parameters.

For capturing location/scale differences, the nonparametric model offered high power in large samples. Parametric models, while potentially having higher power, are more sensitive to violations of distributional assumptions and thus more likely to be rejected when misspecified, especially with large samples. When distributional differences were subtle (e.g., $\Delta\lambda$ in the Wald model), both parametric and non-parametric methods showed a low statistical power. Overall, non-parametric approaches may be preferable, particularly given the risk of misspecifying the parametric model. This is especially relevant in applied settings, where assumptions about the data-generating process are often difficult to justify.

## Simulation 3

While both the PCRM and the DIM include controlled and automatic processes, the meaning of these processes differs between the two models. In the PCRM, control involves accurate processing of the presented target image, whereas in the DIM, control refers to the attempt to overcome prior automatic biases. This difference is relevant when searching for interventions for bias reduction that focus on control-oriented strategies suggested by the PCRM or the DIM (Calanchini et al., 2021; Klauer & Voss, 2008). However, before considering interventions, it is crucial to determine the underlying model first.

Given the lack of systematic comparisons of parametric and nonparametric RT-MPT extensions, it is unclear which approach is better suited for selecting between non-nested models that differ in assumptions that manifest in the distribution of RTs, such as the order of processes. As a remedy, Simulation 3 assesses the performance of non-parametric and parametric approaches (Heck & Erdfelder, 2016; Heck et al., 2018) in detecting whether the PCRM or the DIM is the true, data-generating model.

### Simulation conditions

Simulation 3 is based on the same conditions as Simulations 1 and 2, while using the PCRM and the DIM in a fully crossed 2 x 2 design of models used for data generation and fitting. Again, only models that demonstrated adequate fits in Simulation 1 were evaluated.

### Fitting conditions

In Simulations 1 and 2, the DIM was fitted only to data generated by the DIM, whereas the PCRM was fitted only to data generated by the PCRM. In contrast, in Simulation 3, each of the two models is fitted to all simulation conditions. We fitted the same model versions as described in Simulation 1, but only included the shifted Wald, shifted gamma, and shifted lognormal distributions with R1 constraints. This means that we only fixed the nondecision time parameter (which determines the additive shift of RTs in all branches) while freely estimating the other two distributional parameters across different component distributions (see Table 2).

### Performance measures

Since both the PCRM and the DIM were used for data generation and model fitting, non-nested model comparisons were performed. Model recovery was assessed using the Akaike information criterion ($\mathrm{AIC}$). To assess the relative degree of support for the competing models, we computed AIC weights (wAIC; Burnham & Anderson, 2004; Wagenmakers & Farrell, 2004). The weights are based on differences in AIC values, which are normalized to sum to one and can be interpreted as the relative support for each model. Assuming that there are $J$ competing models, the $wAI{C}_{j}$ of the $j$-th model is calculated as

2 $$wAI{C}_{j}=\frac{{e}^{-\frac{1}{2}(AI{C}_{j}-AI{C}_{min})}}{\sum_{i=1}^{J}{e}^{-\frac{1}{2}(AI{C}_{i}-AI{C}_{min})}}$$

where $AI{C}_{min}$ is the smallest $AIC$ value among all considered models. In the results, we always report the $wAIC$ in favor of the DIM (${wAIC}_{DIM}$), averaged across all simulation replications, which represents the wAIC for selecting the DIM model. Values around .50 indicate an overall lack of preference in model selection, while values above .50 imply a general preference for the DIM and values below .50 suggest a preference for the PCRM. Our aim is to analyze whether, as the effect sizes for differences in RT distributions increase, ${wAIC}_{DIM}$ tends towards 1 when the true model is the DIM and towards 0 when it is the PCRM.

### Results

**Fig. 9:**
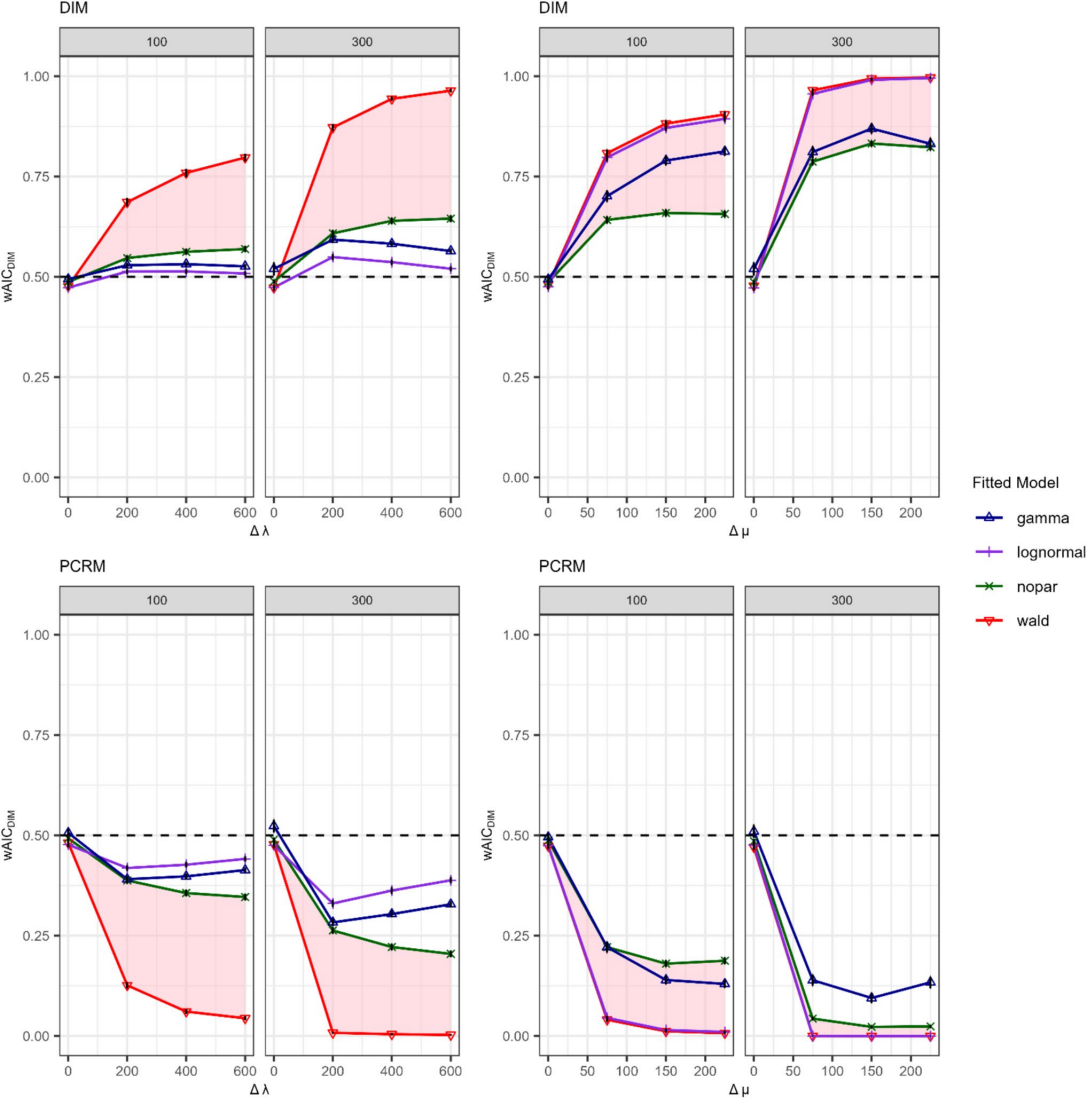
Non-nested model comparison with AIC weights in favor of the DIM relative to the PCRM. *Note*. Average $AIC$ weights larger than.50 favor the DIM model, whereas values below .50 favor the PCRM. Differences between process-specific RT distributions were manipulated via the location/scale parameter ∆µ (*two right columns*) or the shape parameter ∆$\lambda$ (*two left columns*). The labels DIM (*top row*) and PCRM (*bottom row*) refer to the data-generating process. The title of each plot denotes the number of observations per tree (100 or 300).

The results shown in Fig. 9 illustrate the average ${wAIC}_{DIM}$ weights for each fitted model under varying effect sizes of the ${{\Delta} {\mu}}$ and $\Delta \lambda$ manipulations for sample sizes of 100 and 300. In scenarios where there are no differences between the latent RT distributions of the different branches (${{\Delta} {\mu}} =0$ and $\Delta\lambda=0$), we can see that the mean ${wAIC}_{DIM}$ is around .50 as expected. This indicates that the probability of selecting between the two identical models is at the chance level. This pattern was consistent irrespective of whether the data were generated by the DIM (top row) or by the PCRM (bottom row). This shows that, in the absence of discrepancies in process-specific RT distributions, it is not possible to distinguish the two models. Notably, this pattern was observed for all fitted models.

When manipulating the location/scale parameter ${{\Delta} {\mu}}$, the ${wAIC}_{DIM}$ weights for the shifted Wald model (red lines) showed poorer sensitivity for recovering the DIM (two right columns, top row) compared to recovering the PCRM (two right columns, bottom row), particularly with small sample sizes ($n=100$). However, with a larger sample size of 300, the average ${wAIC}_{DIM}$ weights were close to 1 for the DIM and close to 0 for the PCRM (two right panels), indicating almost perfect model recovery. When manipulating ${{\Delta} {\mu}}$ (two right columns), fitting a non-parametric model resulted in a reduction in sensitivity compared to fitting the data-generating, shifted Wald model, as highlighted by the red area in Fig. 9. Nonetheless, it is noteworthy that with larger sample sizes ($n=300$), we achieved a relative probability of correctly selecting the DIM model of 80% while we almost had a probability of 100% with the PCRM. For the alternative parametric models, performance depended on the distributional assumptions. The gamma model performed poorly when manipulating ${{\Delta} {\mu}}$, whereas the lognormal model had a high sensitivity for recovering the true model for all conditions of sample sizes and effect sizes.

For manipulations of the shape parameter $\Delta \lambda$ (two left columns), results for fitting the shifted Wald model were similar to those of manipulating ${{\Delta} {\mu}}$. Power was larger for recovering the PCRM than the DIM, while this difference diminished with larger sample sizes. The nonparametric model (green line) showed particularly poor performance, as illustrated by the area highlighted in red, which is significantly larger for the $\Delta \lambda$ than the ${{\Delta} {\mu}}$ manipulation. Regarding the sensitivity of misspecified parametric approaches, both the gamma and lognormal models had a very low model-recovery performance, and in fact performed worse at larger effect sizes $(\Delta\lambda>200)$.

### Discussion

Simulation 3 shows that, as expected, the correctly specified parametric models achieve higher model-recovery performance than the non-parametric approach. Therefore, if the underlying distributional family were known, the parametric model would naturally be the preferred option. However, we usually do not know the underlying distribution of the data, and as seen in Simulation 3, the parametric approach is highly sensitive to incorrect distributional assumptions. For instance, when RTs were generated by a shifted Wald distribution and differences stem from changes in the $\mu$ parameter, the lognormal model outperformed other models. Conversely, the gamma model had a low sensitivity for finding both location/scale and shape manipulations. In scenarios where subtle changes in the shape of the Wald distribution were introduced via the $\lambda$ parameter, all models – except the correctly specified one – experienced a considerable loss of power. In such cases of misspecification, the non-parametric approach emerged as the best option.

The allure of the non-parametric option lies in its robustness against model misspecification. However, this method suffered a significant loss in model-recovery performance under certain conditions. For example, in detecting the DIM model with $n=100$, performance for ${{\Delta} {\mu}}$ dropped up to 26.1%, and for $\Delta\lambda$ up to 28.6%. That is, it was particularly vulnerable to small sample sizes and substantially lost its ability to accurately recover the true model when RT differences were subtle, or when the distributional manipulations were so specific to a given distribution that the alternative parametric specifications were unable to mimic these changes – as observed with the Wald distribution when modifying the $\lambda$ parameter (see Appendix B).

## General discussion

The present study compared parametric (Heck et al., 2018) and non-parametric (Heck & Erdfelder, 2016) approaches through Monte Carlo simulations to assess their performance across various conditions. Our analysis explored typical research objectives such as goodness-of-fit tests, validation of assumptions about process-specific RT distributions, and the ability to compare the non-nested PCRM and the DIM models for the weapon identification task (Payne, 2001; Rivers, 2017).

A key distinction between the parametric and non-parametric approaches is that parametric models are inherently more restrictive due to their explicit distributional assumptions. As a result, they are also more parsimonious in their predictions and easier to reject empirically. Overall, our results indicate that the non-parametric models are robust and provide adequate goodness-of-fit across various conditions, although they require larger sample sizes to maintain sufficient statistical power to detect RT differences. In contrast, parametric models are highly sensitive to assumptions about the distribution of process-specific RTs and heavily depend on the validity of these assumptions. Not all right-skewed distributions are equally suitable for modeling RT data. Specifically, for RTs simulated by a shifted Wald distribution, only the lognormal distributions showed acceptable fits for changes in the location/scale parameter. This underscores the need to choose an appropriate distributional family that matches the nature of RTs assumed for the branches of the MPT model. For example, Appendix B, Figure B1 shows that manipulating the $\mu$ parameter of the lognormal distribution closely mimics the effects of manipulating the $\mu$ parameter of the Wald distribution. Moreover, it was important to allow the distributional parameters that characterize the fitted, process-specific RT distributions to freely vary for the different components in order to allow for a sufficiently close match with the true, data-generating distributions.

### Recommendations

Our results show that choosing either the parametric or the nonparametric approach has significant methodological and practical consequences in terms of several factors. Sample size impacts the performance of the non-parametric approach. The expected magnitude of effect sizes and, crucially, the nature of these differences, whether they are due to changes in scale, location, or shape, must be taken into account when selecting a modeling approach and its underlying assumptions. It is worth emphasizing that, to enhance the generalizability of our recommendations, Appendix C extends our simulations by incorporating alternative data-generating distributions. These supplementary simulations provide additional support and reinforce both the conclusions and the recommendations we present below.

As to the question of which approach to choose in practice, we cannot give a simple, general answer since each method has its strengths and weaknesses. However, some recommendations can be made. Although a large sample size is preferable for both procedures, this requirement is more critical for the non-parametric approach^5^. As for the parametric approach, although it may require sacrificing the parsimony of the models, one should ensure that the constraints on the parameters do not impede the flexibility of the fitted RT distributions, since the procedure is very sensitive to misspecifications. That is, we should estimate two or more free parameters for each process-specific RT distribution rather than assuming that differences in latent RT distributions can a priori be attributed to a single parameter (e.g., by estimating different location/scale parameters while fixing the shape parameter to be identical across RT distributions). Also, to ensure robustness of the results, one may consider fitting multiple models with different distributional families. Thereby, one can test for differences in latent RT distributions while making different auxiliary assumptions about the type of these changes (e.g., in terms of location, scale, shape, etc.).

Regarding the previous point, we would like to emphasize that the subtle manipulations of the shape of RT distributions via the $\lambda$ parameter of the Wald distribution were not well captured by any model other than the true model itself. This outcome was expected, as our analysis of the parameterizations across different distributions (see Appendix B) revealed that it is not possible to achieve a perfect equivalence of distributional moments by capturing manipulations of $\lambda$ in the Wald model by parameters of the other distributions. That is, when fine-grained distributional changes are introduced, only a parametric model that closely mimics the true data-generating process can effectively detect them, as the non-parametric alternative is generally much less sensitive to subtle shape changes (see additional supporting evidence in Appendix C; for example, the lognormal model was the only alternative able to detect $\Delta \sigma$ manipulations in ex-Gaussian data). This is noteworthy because it suggests that if our goal is to detect subtle distributional shifts, we may need to test several variants across different parametric families to identify a model specification capable of accounting for the specific idiosyncrasies involved. Conversely, if our research question does not demand this level of detail, the non-parametric approach might be preferable due to its robustness. This trade-off ultimately depends on the study’s objectives. A compelling example can be found in the literature on attention-deficit/hyperactivity disorder, where individuals are frequently reported to exhibit slower average response times in several tasks. However, when these RT differences are modeled using an ex-Gaussian distribution (Bella-Fernández et al., 2024; Gu et al., 2013; Hervey et al., 2006; Osmon et al., 2018; Whelan, 2008), the findings reveal that such differences are not uniformly distributed across trials. Instead, they primarily emerge in a subset of responses likely associated with attentional lapses. That is, in order to detect certain highly specific patterns (e.g., overly slow RTs in a small number of trials), it may be necessary to fit parametric models that can precisely capture such changes in the shape of RT distributions.

To apply the non-parametric method, one needs to decide how many bins to use and how to set the boundaries to categorize RTs into bins. We believe that model parsimony should be balanced with the type of mechanisms we aim to uncover. For our example, we used only two bins (relatively fast and relatively slow) because the verbal PCRM model describes controlled processes as slower than automatic ones. Therefore, it is parsimonious and sufficient to use only two bins to test whether automatic responses have a higher probability of being fast than controlled responses. Regarding the boundaries for categorizing RTs into bins, individual differences play a significant role, as what is considered a “fast” response usually varies across participants. To address this, we set boundaries separately for each individual using data-dependent RT criteria (Heck & Erdfelder, 2016). To illustrate how we discretized continuous RTs and fitted the PCRM and DIM models using a non-parametric approach with real-world data, Appendix A provides reproducible code for analyzing the data from Amon and Holden (2016).^6^ Other methods, such as an initial calibration phase in the experiment, could also be considered to identify appropriate boundaries to discretize RTs into bins. It can happen that certain boundaries result in bins with few or no observations, posing problems for parameter estimation and χ^2^ tests since asymptotic properties may not hold (Langeheine et al., 1996). Also, note that the data-generating process may lead to changes in the distributional shape that are not reflected by the binning structure when using only two RT bins based on a specific binning criterion, such as the geometric mean RT. In other words, differences in the underlying RT distributions may not affect the relative proportion of fast responses. In such cases, the non-parametric approach will not be able to detect any changes in RTs, and a well-specified parametric model may be required to identify them. Alternatively, modifying the binning procedure or increasing the number of bins could improve sensitivity to detect subtle distributional changes. This could be one possible explanation for the lower power of the two-bin non-parametric approach when detecting small or subtle effects.

Beyond these recommendations, we would like to emphasize that there is always a trade-off between parametric and non-parametric approaches, and the choice ultimately depends on the researcher’s goals when fitting a model. A parametric RT-extended MPT model that is misspecified (see, for instance, Simulation 1) will typically show poor fit to the data, and fitting such models requires strong assumptions about the underlying distributions. Frequently, however, there is insufficient evidence to determine the appropriate parametric form, in which case non-parametric models – despite their shortcomings – offer more robustness against misspecification.

That said, we still believe parametric models remain valuable because they allow researchers to test the validity of theoretical assumptions and to move beyond purely descriptive accounts of fast or slow responses. For example, Klauer and Kellen (2018) explicitly assume that latent processing times in MPT models follow exponential distributions and motor times truncated Gaussian distributions, which together yield an ex-Gaussian if only one process is involved. This framework not only evaluates whether the ex-Gaussian adequately describes the data but also makes it possible to investigate which experimental manipulations selectively influence different components of the distribution, thereby providing deeper insights into the underlying cognitive processes.

Further investigation is necessary for addressing challenges in extending MPT models with RT data. While a first introduction regarding the application of MPT-RT models in social cognition was recently published (Wilson et al., 2025), best practices and specific recommendations should be compiled into a user-friendly tutorial to guide researchers. Such a tutorial could be useful in promoting the wider use of RT-MPT models and ensuring robustness of results in light of the many degrees of freedom researchers have in modeling.

## Limitations and future research

A limitation of our simulations is that we only compared two specific MPT models, the DIM and the PCRM. Other MPT model structures may require different effect sizes to achieve the same level of statistical power. As in any simulation study, our findings about which parametric assumptions are optimal are specific to the implemented conditions and cannot universally be generalized, as such assumptions should be considered on a study-by-study basis.

Furthermore, alternative parametric approaches such as the hierarchical RT-MPT model by Klauer and Kellen (2018) and the recently introduced MPT-diffusion model by Klauer et al. (2024) were not assessed in our study. Comparing these models could yield valuable insights. The hierarchical RT-MPT model approach (Klauer & Kellen, 2018) introduces additional distributional assumptions about processing and motor times—a crucial consideration given the sensitivity of parametric models against misspecification observed in our simulations. Specifically, the RT-MPT model approach assumes that motor times follow truncated normal distributions while processing times follow exponential distributions. This modeling approach increases precision and is likely to come with a higher sensitivity, but it may lead to incorrect conclusions when the actual distributions of process-specific RTs differ too much from the assumptions. In the present study, we tested the effect of such a misspecification by generating data with the shifted Wald while fitting other distributions, such as the ex-Gaussian (which resembles the RT-MPT model approach for latent states with a single processing time). The potentially large impact of misspecified distributions underscores the need for adaptations of the *rtmpt* package (Hartmann et al., 2020) to encompass more distributional families and thus accommodate RT distributions across a wider range of scenarios.

It is important to note that accounting for hierarchical structures (e.g., trials nested in persons) may offer additional advantages that were not considered in our comparison between parametric and nonparametric MPT models. One key benefit of hierarchical models concerns small sample sizes per subject. While the nonparametric approach by Heck and Erdfelder (2016) can also be implemented within a hierarchical framework (Heck & Erdfelder, 2017; Schmidt et al., 2023), a current limitation of the parametric approach proposed by Heck et al. (2018) is the need to fit individual-level data separately. According to Chechile (2009), hierarchical MPT models are more robust to small sample sizes than individual-level analyses. Therefore, future comparisons that incorporate hierarchical modeling may yield different results than those reported in the present study. For instance, our finding that non-parametric models exhibit substantial power loss at the individual level with smaller sample sizes might be alleviated under a hierarchical structure. Thus, a comparison of hierarchical parametric and non-parametric RT-MPT approaches remains unexplored and would be of great interest for future research.^7^

Another important aspect to consider when fitting models is *model mimicry*. Instead of using models assuming discrete latent states, such as MPT models, other types of cognitive models have been developed for the joint analysis of continuous and discrete variables. Among these, the diffusion model (Ratcliff, 1978; Ratcliff et al., 2016) stands out as a specific class of evidence-accumulation models often used for analyzing choice decision tasks such as the WIT (see, for example, Todd et al., 2021). Given the existence of different ways to model unobserved processes, a potential limitation of any modeling approach is that selecting an incorrect model—i.e., model misspecification—could lead to erroneous substantive interpretations. Thus, the results of MPT-RT modeling are always conditional on the assumption that the underlying MPT model is appropriate. Parameter estimates do not have a clear, valid interpretation if the MPT model itself is misspecified (e.g., if a diffusion model generates the data). Nevertheless, some models may be sufficiently flexible to fit empirical data well. For instance, the true data-generating process might be a diffusion model, yet a sufficiently flexible MPT-RT model could still produce an acceptable fit. However, even if an RT-MPT model is not in line with the data-generating distribution, it might be a useful tool for measurement and theory testing (Heck & Erdfelder, 2017). We thus consider it necessary to fit MPT-RT models with sufficiently flexible RT distributions to ensure that the core MPT mixture assumptions can still be tested and evaluated. Striking an appropriate balance between flexibility and parsimony regarding the number of latent processes and RT component distributions is crucial, and further exploration of this issue remains an important line of future research.

In our study, we address one potential challenge researchers may face when extending MPT models with RTs. Specifically, in parametric modeling, it is necessary to assume a distribution that accounts for task-specific factors that influence RT data, such as the emphasis on accuracy or speed, task complexity, anticipation, and experimental manipulations. More importantly, RT data should provide insight into the mechanics and explanations of cognitive processes (Van Zandt, 2002), which vary across experiments. Therefore, a flexible parametric approach like the one proposed by Heck et al. (2018) is particularly valuable, as it can accommodate a variety of distributions commonly used for RT data, such as the shifted Wald, Weibull, gamma, or ex-Gaussian (Cousineau et al., 2004; Heathcote et al., 1991; Luce, 1986; Palmer et al., 2011; Ratcliff & Murdock, 1976; Van Zandt, 2002). However, since the true underlying distribution is unknown and there may be situations where a strong theoretical understanding of the data-generating mechanisms is lacking, it may be necessary to use a non-parametric approach.

## Conclusions

Overall, our simulation study guides applied researchers in choosing between parametric and non-parametric approaches when extending MPT models in order to include RTs or other continuous variables. We hope that this will spur future research based on both response categories and continuous variables to conduct more informative and comprehensive tests and to exploit the potential of MPT models for disentangling cognitive processes underlying human behavior.

## Supplementary Information

Below is the link to the electronic supplementary material.

Supplementary file1 (DOCX 3227 KB)

## Data Availability

All simulated data and R code for data generation, data analysis, auxiliary functions, and visualizations are available at the Open Science Framework, https://osf.io/54mdq/.

## Footnotes

1 The authors originally used the terms *“fast”* and *“slow”* to refer to responses that are faster or slower *relative* to each participant’s overall RT distribution across all response categories. To classify responses, a log-normal approximation was applied: individual RTs were log-transformed, quantiles were calculated, and then these were converted back to the original scale to define person-specific cut-offs for categorizing responses. This approach follows recommendations based on simulation studies (see log-normal approximation procedure and simulations in Heck & Erdfelder, 2016).

2 For an example of how to fit the DIM and PCRM using a non-parametric approach with real-world data, see the reproducible code provided in Appendix A. In this example, we start from the raw data provided by Amon & Holden (2016), which includes over 1000 trials per subject in the weapon identification task, and fit both the PCRM and DIM models. The original data and scripts can be found in our OSF repository (https://osf.io/54mdq/).

3 There is also a recent proposal by Klauer et al. (2024) that combines MPT models and diffusion models.

4 The parametric restrictions of Model_eq_ were established by fixing the *d* and *s* parameters of each model to zero (see Table 2).

5 In fact, given sufficient data, any parametric model that is not identical to the data-generating one would eventually be statistically rejected. However, this might not be desirable. The true data-generating distribution is usually unknown, it may not perfectly correspond to a parametric distribution, and testing all plausible alternatives is often impractical. Also, the objective may not necessarily be to identify the true data-generating model, but rather to distinguish meaningful and spurious patterns. Ultimately, the relevance of misspecifications will depend on the specific goals of the research question.

6 The non-parametric approach produced results consistent with earlier research favoring the DIM model (Laukenmann et al., 2023).

7 Comparisons withing MPT estimation methods have been conducted in Singmann et al., (2024). Although the divergence between them was, on average, small, hierarchical methods showed the best results. However, these comparisons did not include extensions of MPT models that incorporate continuous variables, such as RTs.
