# Supplementary material for: Extensions of multinomial processing tree models for continuous variables: A simulation study comparing parametric and non-parametric approaches
Source: Behav Res Methods. 2025 Dec 8;58(1):22. doi: 10.3758/s13428-025-02896-9 (PMC12686110; doi:10.3758/s13428-025-02896-9)
Supplement: Supplementary file 1 — Supplementary file1 (DOCX 3227 KB) [file 13428_2025_2896_MOESM1_ESM.docx]

**Appendix A. Fits for the data by Amon and Holden (2016)**

# 1 Overview

This appendix documents the R code used to analyze the data from Amon & Holden (2016) using the MPT-RT (Heck & Erdfelder, 2016) non-parametric modeling framework. The entire procedure is explained step-by-step, following the script presented below.

## 1.1 . Load functions

extract_estimates <- function(fit) {
 setNames(fit$parameters$estimates, rownames(fit$parameters)) |>
 t() |> as.data.frame()
}

recateg.Bbin <- function(data, b = 2, groupby = "x") {
 y_break <- exp(quantile(log(data$y), seq(0, 1, length.out = b + 1)))
 data$y_cat <- cut(data$y, breaks = y_break, include.lowest = TRUE,
 labels = 1:b, na.rm = TRUE)
 cat <- c("cr", "fa", "hit", "miss")
 tree <- c(rep(c("bt1", "bt2", "wt1", "wt2"), times=2),
 rep(c("bg1", "bg2", "wg1", "wg2"), times=2))
 mptrt_categories <- paste0(rep(cat, each=4), "_", tree)
 data$mptrt_category <- factor(paste0(data[[groupby]], data$y_cat),
 levels = mptrt_categories)
 frequencies <- c(table(data$mptrt_category))
 names(frequencies) <- mptrt_categories
 frequencies <- frequencies[paste0(rep(c("hit_bg", "miss_bg",
 "cr_bt", "fa_bt",
 "hit_wg", "miss_wg",
 "cr_wt", "fa_wt"),
 each=2), 1:2)]
 return(list(frequencies = frequencies, y_break = y_break))
}

## 1.2 . Specify Study Conditions

input_dir <- paste0(getwd(), "/Ctl")
files <- list.files(input_dir, pattern = "\\.csv$", full.names = TRUE)

approach <- "nopar"
model_dir <- file.path(normalizePath(file.path(getwd(), "..")), "model_files")
m.dim.file <- file.path(model_dir, paste0("m.dim_", approach, ".txt"))
m.pcrm.file <- file.path(model_dir, paste0("m.pcrm_", approach, ".txt"))

## 1.3 Fit Models in Parallel

ini_time <- Sys.time()

n.cores <- parallel::detectCores(logical = FALSE) - 1
cl <- parallel::makeCluster(n.cores, type = "SOCK")
doParallel::registerDoParallel(cl, cores = n.cores)

res <- foreach::foreach(i = 1:length(files),
 .packages = c("MPTinR", "dplyr", "readr"),
 .combine = bind_rows,
 .inorder = FALSE
) %dopar% {
 try({
 df <- read_csv(files[i]) %>%
 filter(!is.na(race), !is.na(tool)) %>%
 mutate(
 tree = paste(race, tool, sep = "_"),
 x = case_when(
 race == "black" & tool == "gun" & Correct ~ "hit_bg",
 race == "black" & tool == "gun" & !Correct ~ "miss_bg",
 race == "black" & tool == "tool" & Correct ~ "cr_bt",
 race == "black" & tool == "tool" & !Correct ~ "fa_bt",
 race == "white" & tool == "gun" & Correct ~ "hit_wg",
 race == "white" & tool == "gun" & !Correct ~ "miss_wg",
 race == "white" & tool == "tool" & Correct ~ "cr_wt",
 race == "white" & tool == "tool" & !Correct ~ "fa_wt",
 TRUE ~ NA_character_
 ),
 x = factor(x, levels = c(
 "hit_bg", "miss_bg", "cr_bt", "fa_bt",
 "hit_wg", "miss_wg", "cr_wt", "fa_wt"
 )),
 y = RT
 ) %>%
 select(tree, x, y, block = Block)

 data.bin <- recateg.Bbin(df, b = 2, groupby = "x")
 fit.dim <- fit.mpt(data.bin$frequencies, m.dim.file, output = "full")
 fit.pcrm <- fit.mpt(data.bin$frequencies, m.pcrm.file, output = "full")
 fit.dim.rest <- fit.mpt(data.bin$frequencies, m.dim.file,
 restrictions.filename = list("d1=d2=1"), output = "full")
 fit.pcrm.rest <- fit.mpt(data.bin$frequencies, m.pcrm.file,
 restrictions.filename = list("d1=d2=1"), output = "full")

 output <- c(
 id=i,
 file = basename(files[i]),
 nested.dim = test_nested_nopar(fit.dim, fit.dim.rest),
 nested.pcrm = test_nested_nopar(fit.pcrm, fit.pcrm.rest),
 nonnested = test_nonnested_nopar(fit.dim, fit.pcrm),
 nonnested.rest = test_nonnested_nopar(fit.dim.rest, fit.pcrm.rest),
 gof.dim = fit.dim$goodness.of.fit,
 gof.pcrm = fit.pcrm$goodness.of.fit,
 gof.dim.rest = fit.dim.rest$goodness.of.fit,
 gof.pcrm.rest = fit.pcrm.rest$goodness.of.fit,
 par.dim = extract_estimates(fit.dim),
 par.pcrm = extract_estimates(fit.pcrm),
 par.dim.rest = extract_estimates(fit.dim.rest),
 par.pcrm.rest = extract_estimates(fit.pcrm.rest)
 )

 output
 })
}
parallel::stopCluster(cl)
end_time <- Sys.time()
print(end_time - ini_time)

## Time difference of 36.1538 secs

## 1.4 . Plot Results

## 1.4.1 Nested Comparison

alpha <- 0.05

dim_sig <- ifelse(res$nested.dim.prob < alpha, "Significant", "Not significant")
pcrm_sig <- ifelse(res$nested.pcrm.prob < alpha, "Significant", "Not significant")

df_nested <- data.frame(
 Model = rep(c("dim", "pcrm"), each = length(dim_sig)),
 Result = c(dim_sig, pcrm_sig)
)

df_count <- df_nested %>%
 dplyr::group_by(Model, Result) %>%
 dplyr::summarise(Count = dplyr::n(), .groups = "drop")

# Plot
library(ggplot2)
ggplot(df_count, aes(x = Model, y = Count, fill = Result)) +
 geom_bar(stat = "identity", position = "stack", width = 0.6, color = "black") +
 geom_text(aes(label = Count), position = position_stack(vjust = 0.5), size = 4) +
 scale_fill_manual(values = c("Significant" = "black", "Not significant" = "gray60")) +
 labs(title = "Nested Model Comparison (p < 0.05)", x = "Model", y = "Count", fill = "Result") +
 theme_minimal(base_size = 12)


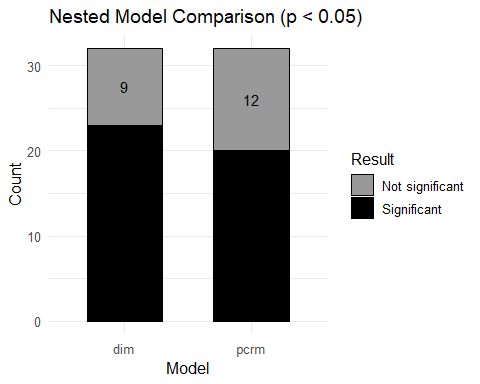


## 1.4.2 Non-nested Comparison

model_choices <- res$nonnested.sel_AIC
model_choices[is.na(model_choices)] <- "dim = pcrm"

df <- as.data.frame(table(model_choices))
colnames(df) <- c("Model", "Count")

ggplot(df, aes(x = Model, y = Count, fill = Model)) +
 geom_bar(stat = "identity", width = 0.6, color = "black") +
 geom_text(aes(label = Count), vjust = -0.4, size = 4) +
 scale_fill_manual(values = c("dim" = "steelblue",
 "pcrm" = "tomato",
 "dim = pcrm" = "gray60")) +
 labs(title = "Non-nested Model Comparison (AIC)",
 x = "Selected Model",
 y = "Count") +
 theme_minimal(base_size = 12) +
 theme(legend.position = "none")


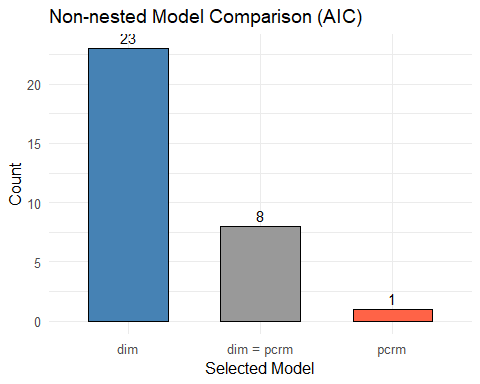


**Appendix B. Parametrization**

In the following, we explain how we selected the free parameters for each distributional family—both in model fitting and simulations—that may be used to (at least partially) account for specific differences between the various data-generating RT distributions. Our selection of the parameterization is based on the implied changes on the densities of the component distributions. Figures B1, B2, and B3 illustrate how certain manipulations of the parameters of different RT distributions can mimic the effects of increasing $\mu$, $\lambda$, and $\delta$ for the shifted Wald distribution. Figure B1 depicts parameters changes that resemble the effect of increasing the location/scale parameter ($\mu$), while Figure B2 illustrates the effect of increasing the scale parameter ($\lambda$). The two figures also show the corresponding, approximately similar manipulations for the other RT distributions. Figure B3 displays how changes of the shift parameter of each distribution result in equivalent effects as changing the non-decision time parameter $\delta$ in the Wald distribution.

**Figure B1.**

*Approximation of the effect of changing the location/scale parameter* $\mu$ *of the shifted Wald distribution*

**
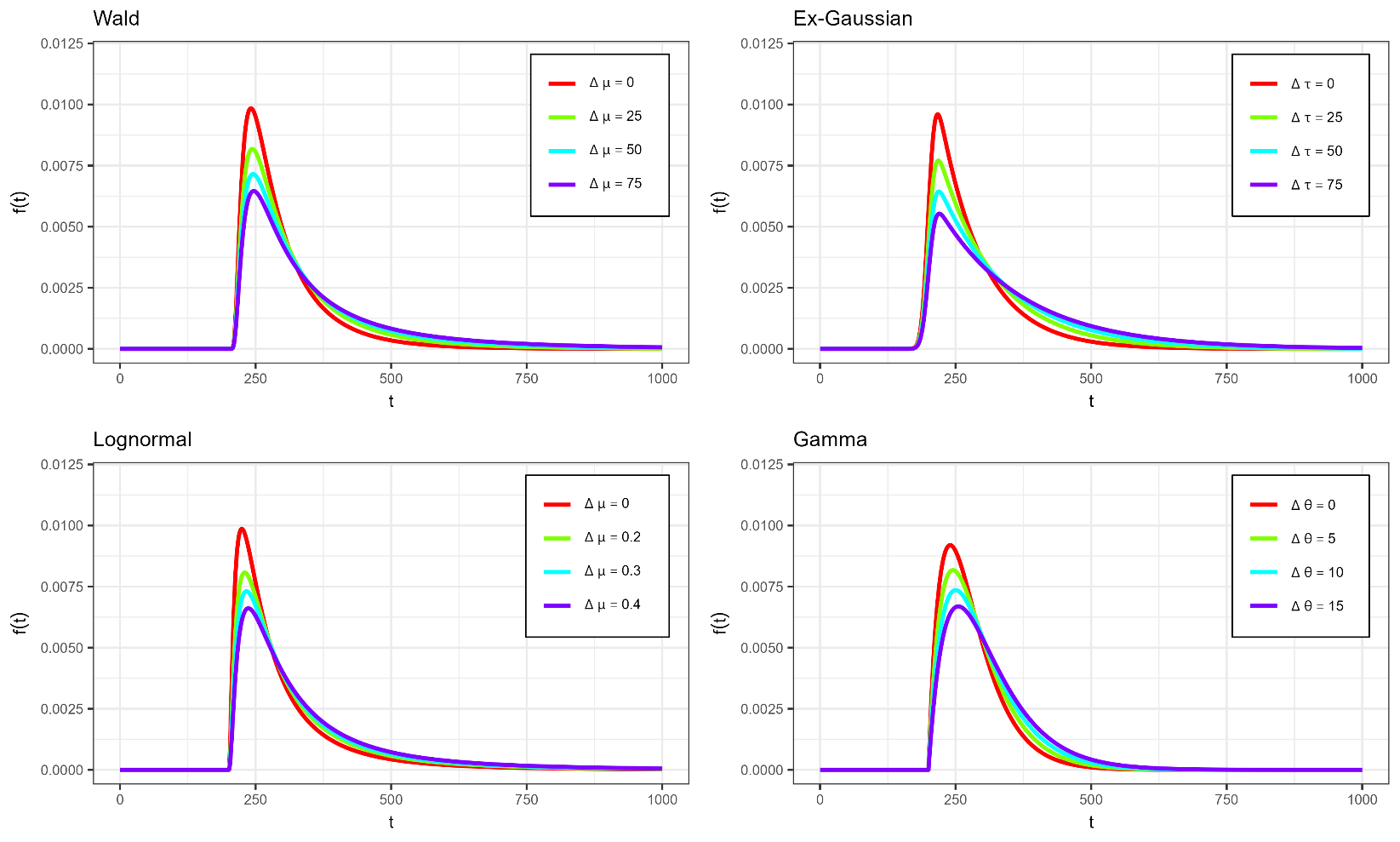
**

**Figure B2.**

*
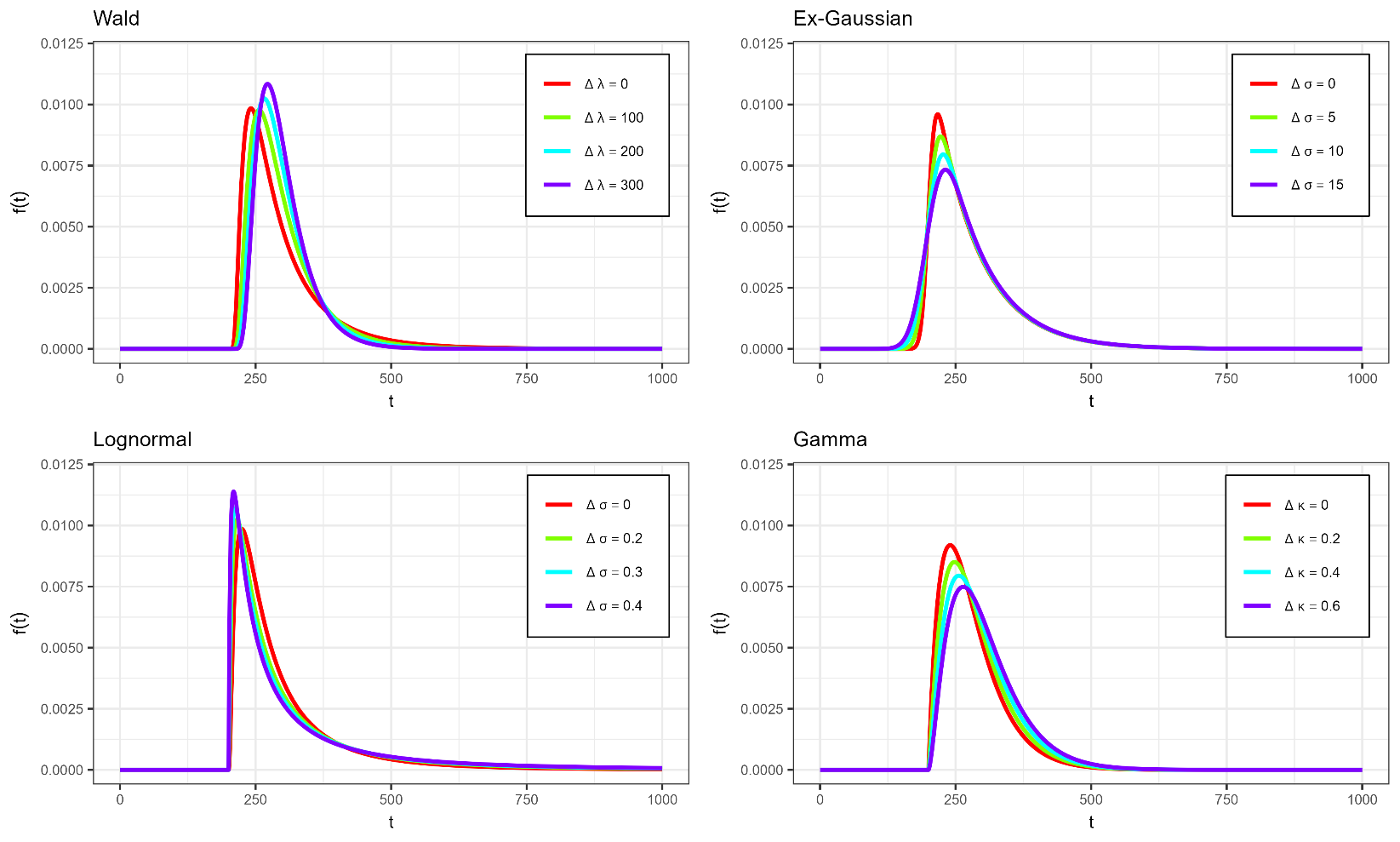
Approximation of the effect of changing the shape parameter* $\lambda$ *of the shifted Wald Distribution*

**Figure B3.**

*
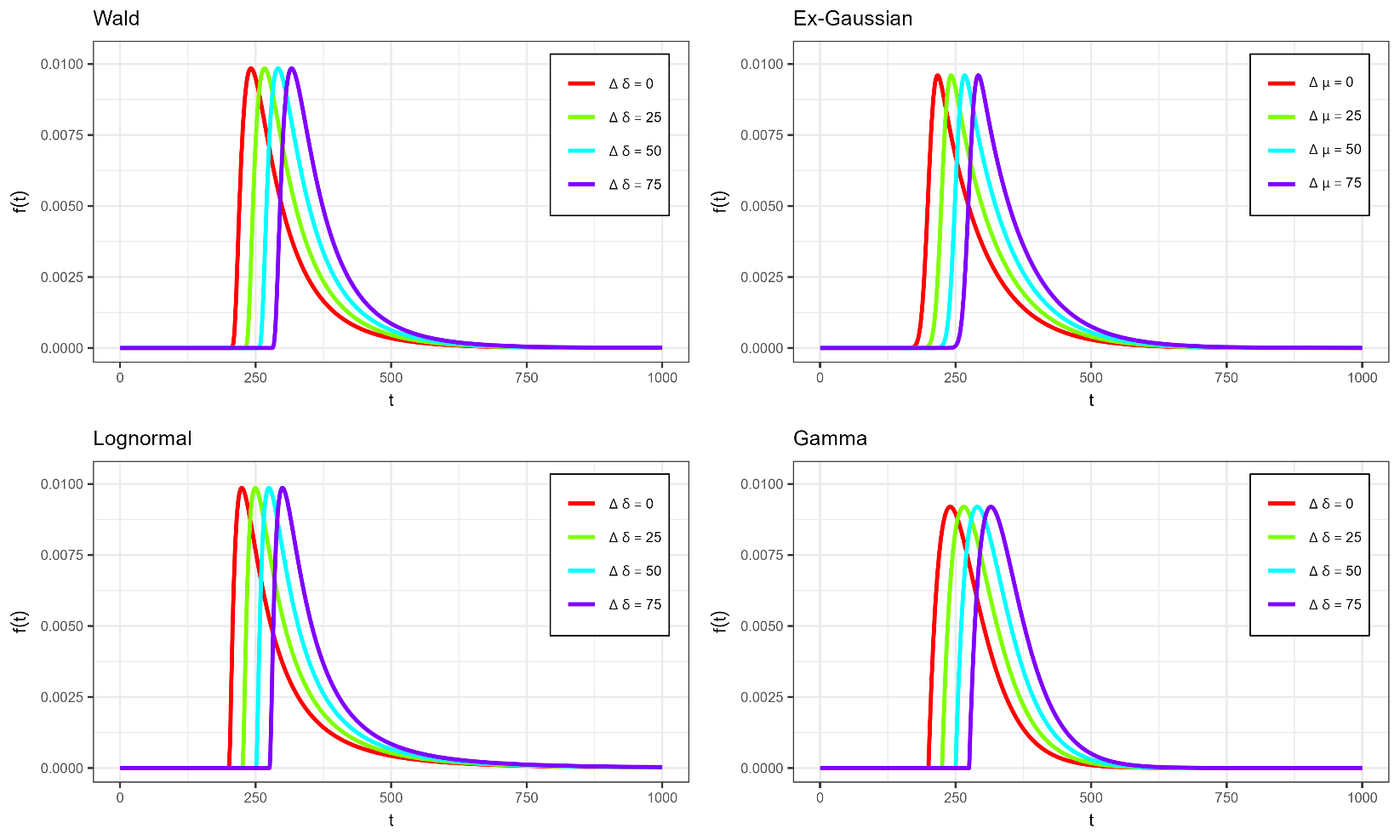
 Equivalence of the effect of changing the shift parameter* $\delta$ *of the shifted Wald Distribution*

The figures above suggest that some parameters function similarly across different distributions. For instance, Figure B3 demonstrates that all distributions possess a parameter capturing additive shifts of the non-decision time for RT distributions. To ensure comparability across distributions, when fitting data using alternative distributions, we decided to fix the non-decision time at 200ms, regardless of the simulation or fitting condition. However, finding equivalent parameterizations for the other parameters is more complex. To better understand the effects of parameter manipulations and to identify approximately equivalent transformations to those induced to the Wald distribution, we assessed how each parameter influences the moments of the RT distribution when a constant is added or subtracted to each parameter^[[1]](#footnote-1)^. Table B1 summarizes how specific parameters affect the mean, variance, and skewness of the distribution.

Since achieving exact equivalence of statistical moments is impossible when manipulating a single parameter alone, we aimed to apply transformations that were at least roughly comparable across distributions. For instance, increasing $\mu$ in the Wald distribution increases both the mean and variance—an effect that can be approximated in the ex-Gaussian by increasing *τ*, in the shifted lognormal by increasing $\mu$, and in the shifted gamma by increasing the scale parameter. In contrast, in the normal distribution, only the mean is varied.

When manipulating $\lambda$ in the shifted Wald distribution, the variance decreases along with a reduction in skewness (see Figure B2). However, this effect on moments cannot be exactly reproduced by manipulating a single parameter in the other distributions. For example, in the ex-Gaussian, increasing *σ* decreases skewness, but unlike the Wald, it increases the variance. In the shifted lognormal, increasing *σ* also increases both skewness and variance, and slightly affects the mean—an outcome that does not match the behavior of the Wald. Alternatively, reducing *σ* in the lognormal to decrease skewness creates another problem: it produces overly narrow distributions, which do not resemble those generated by the Wald, especially for large effect sizes. In the shifted gamma, increasing the shape parameter *k* decreases skewness but simultaneously increases both the variance and the mean, again diverging from the Wald’s behavior. As for the normal distribution—which has zero skewness by definition—we only attempted to approximate the reduction in variance.

These parametrizations were designed to ensure that the effects of parameter restrictions remained reasonably comparable across the different data-generating distributions. However, this goal was notably easier to achieve when manipulating non-decision time or $\mu$ in the Wald distribution than when altering $\lambda$. Nevertheless, we decided to retain the $\lambda$ manipulations in our design to explore more ambiguous scenarios and subtle distributional shifts, thereby allowing to analyze how well parametric and non-parametric models perform under such conditions.

**Table B1.**

*Effects of parameter manipulations on the moments of the RT distribution.*

| Distribution | Parameter | |  | | Mean | | Var | | Skew | |
| --- | --- | --- | --- | --- | --- | --- | --- | --- | --- | --- |
| Shifted Wald | *μ* |  | | ↑ | | ↑ | | ↑ | |  |
|  | *λ* |  | | none | | ↓ | | ↓ | |  |
|  | *δ* |  | | ↑ | | none | | none | |  |
|  |  |  | |  | |  | |  | |  |
|  |  |  | |  | |  | |  | |  |
| Ex-Gaussian | *τ* |  | | ↑ | | ↑ | | ↑ | |  |
|  | *σ* |  | | none | | ↑ | | ↓ | |  |
|  | *μ* |  | | ↑ | | none | | none | |  |
|  |  |  | |  | |  | |  | |  |
|  |  |  | |  | |  | |  | |  |
| Shifted lognormal | *μ* |  | | ↑ | | ↑ | | none | |  |
|  | *σ* |  | | ↑ | | ↑ | | ↑ | |  |
|  | *δ* |  | | ↑ | | none | | none | |  |
|  |  |  | |  | |  | |  | |  |
|  |  |  | |  | |  | |  | |  |
| Normal | *μ* |  | | ↑ | | none | | none | |  |
|  | *σ* |  | | none | | ↑ | | none | |  |
|  |  |  | |  | |  | |  | |  |
|  |  |  | |  | |  | |  | |  |
| Shifted gamma | $\theta$ |  | | ↑ | | ↑ | | none | |  |
|  | *k* |  | | ↑ | | ↑ | | ↓ | |  |
|  | *δ* |  | | ↑ | | none | | none | |  |

*Note.* Effects of parameter manipulation on distribution moments. This table summarizes the impact of larger parameter values on the mean, variance, and skewness of different distributions. The modifications aim to produce visual similarities to the effects observed in the Wald distribution; however, exact equivalence of moments cannot be achieved by adjusting a single parameter in isolation.

Overall, this search for equivalences and approximations between parameters forms the basis of the chosen parameterization of RT distributions for data generation (see Appendix C) and model fitting (see Model Fitting section, p. 17–19), aiming to ensure that transformations remain comparable across different PDFs.

**Appendix C. Results with other data-generating distributions**

To assess whether our conclusions are specific to generating data with the Wald distribution, we replicated the simulation in the main text using different data-generating distributions . Specifically, we generated data using the ex-Gaussian and lognormal distribution under conditions similar to those applied to the Wald distribution in the main text. To avoid making this study excessively long and repetitive, we summarize the main outcomes under selected simulation conditions. We again divided the simulations into three major sections: one to evaluate goodness-of-fit, another to study nested comparisons, and a third to assess non-nested comparisons. We refer to these as Simulation C1, Simulation C2, and Simulation C3, respectively.

**Simulation Conditions**

For all simulation conditions, we aimed to maintain similar fitting conditions as those presented in the main text. The settings were identical to the main simulation except otherwise noted. Regarding the continuous distributions, simulated RTs following an ex-Gaussian distribution were characterized by the parameters $\mu$, $\tau$, and $\sigma$. For data generated by the shifted lognormal distribution, the parameters were $\mu, \sigma$, and $\delta$. Tables C1 and C2 present the chosen parameter values $\boldsymbol{\eta}$ for the ex-Gaussian and lognormal models, respectively, as a function of the manipulation of the effect size of inter-branch differences and the nature of these differences (scale and shape differences in the Wald distribution), for both the PCRM and DIM models. In Table B1 (Appendix B), we analyze the role of each parameter and its relationship with the scale and shape differences manipulated for data generation under the Wald distribution.

**Table C1.**

*Parameters* ***η*** *of the ex-Gaussian distribution used for simulating RT data.*

| Effect size |  |  | PCRM | | | | | |  | DIM | | | | | |
| --- | --- | --- | --- | --- | --- | --- | --- | --- | --- | --- | --- | --- | --- | --- | --- |
|  |  |  | Free *η* | | |  | Fixed *η* | |  | Free *η* | | |  | Fixed *η* | |
|  |  |  | $\sigma_{A}$ | $\sigma_{Ct}=\sigma_{A}+\Delta\sigma$ | $\sigma_{Cg}=\sigma_{A}+2\Delta\sigma$ |  | $\tau$ | $\mu$ |  | $\sigma_{\left( 1-C \right)}$ | $\sigma_{C+}=\sigma_{(1-C)}+\Delta\sigma$ | $\sigma_{C-}=\sigma_{(1-C)}+2\Delta\sigma$ |  | $\tau$ | $\mu$ |
| $\Delta\sigma$ | 0 |  | 10 | 10 | 10 |  | 80 | 200 |  | 10 | 10 | 10 |  | 80 | 200 |
|  | 5 |  | 10 | 15 | 20 |  | 80 | 200 |  | 10 | 15 | 20 |  | 80 | 200 |
|  | 10 |  | 10 | 20 | 30 |  | 80 | 200 |  | 10 | 20 | 30 |  | 80 | 200 |
|  | 15 |  | 10 | 25 | 40 |  | 80 | 200 |  | 10 | 25 | 40 |  | 80 | 200 |
|  |  |  |  |  |  |  |  |  |  |  |  |  |  |  |  |
|  |  |  | $\tau_{A}$ | $\tau_{Ct}=\tau_{A}+\Delta\tau$ | $\tau_{Cg}=\tau_{A}+2\Delta\tau$ |  | $\sigma$ | $\mu$ |  | $\tau_{\left( 1-C \right)}$ | $\tau_{C+}=\tau_{(1-C)}+\Delta\tau$ | $\tau_{C-}=\tau_{(1-C)}+2\Delta\tau$ |  | $\sigma$ | $\mu$ |
| $\Delta\tau$ | 0 |  | 80 | 80 | 80 |  | 10 | 200 |  | 80 | 80 | 80 |  | 10 | 200 |
|  | 25 |  | 80 | 105 | 130 |  | 10 | 200 |  | 80 | 105 | 130 |  | 10 | 200 |
|  | 50 |  | 80 | 130 | 180 |  | 10 | 200 |  | 80 | 130 | 180 |  | 10 | 200 |
|  | 75 |  | 80 | 155 | 230 |  | 10 | 200 |  | 80 | 155 | 230 |  | 10 | 200 |

**Table C2.**

*Parameters* ***η*** *of the lognormal distribution used for simulating RT data.*

| Effect size |  |  | PCRM | | | | | |  | DIM | | | | | |
| --- | --- | --- | --- | --- | --- | --- | --- | --- | --- | --- | --- | --- | --- | --- | --- |
|  |  |  | Free *η* | | |  | Fixed *η* | |  | Free *η* | | |  | Fixed *η* | |
|  |  |  | $\sigma_{A}$ | $\sigma_{Ct}=\sigma_{A}+\Delta\sigma$ | $\sigma_{Cg}=\sigma_{A}+2\Delta\sigma$ |  | $\mu$ | $\delta$ |  | $\sigma_{\left( 1-C \right)}$ | $\sigma_{C+}=\sigma_{(1-C)}+\Delta\sigma$ | $\sigma_{C-}=\sigma_{(1-C)}+2\Delta\sigma$ |  | $\mu$ | $\delta$ |
| $\Delta k$ | 0 |  | 1 | 1 | 1 |  | 4.2 | 200 |  | 1 | 1 | 1 |  | 80 | 200 |
|  | 0.2 |  | 1 | 1.2 | 1.4 |  | 4.2 | 200 |  | 1 | 1.2 | 1.4 |  | 80 | 200 |
|  | 0.3 |  | 1 | 1.3 | 1.6 |  | 4.2 | 200 |  | 1 | 1.3 | 1.6 |  | 80 | 200 |
|  | 0.4 |  | 1 | 1.4 | 1.8 |  | 4.2 | 200 |  | 1 | 1.4 | 1.8 |  | 80 | 200 |
|  |  |  |  |  |  |  |  |  |  |  |  |  |  |  |  |
|  |  |  | $\mu_{A}$ | $\mu_{Ct}=\mu_{A}+\Delta\mu$ | $\mu_{Cg}=\mu_{A}+2\Delta\mu$ |  | $\sigma$ | $\delta$ |  | $\mu_{\left( 1-C \right)}$ | $\mu_{C+}=\mu_{(1-C)}+\Delta\mu$ | $\mu_{C-}=\mu_{(1-C)}+2\Delta\mu$ |  | $\sigma$ | $\delta$ |
| $\Delta\mu$ | 0 |  | 4.2 | 4.2 | 4.2 |  | 1 | 200 |  | 4.2 | 4.2 | 4.2 |  | 80 | 200 |
|  | 25 |  | 4.2 | 29.2 | 54.2 |  | 1 | 200 |  | 4.2 | 29.2 | 54.2 |  | 80 | 200 |
|  | 50 |  | 4.2 | 54.2 | 104.2 |  | 1 | 200 |  | 4.2 | 54.2 | 104.2 |  | 80 | 200 |
|  | 75 |  | 4.2 | 79.2 | 154.2 |  | 1 | 200 |  | 4.2 | 79.2 | 154.2 |  | 80 | 200 |

The parameter values for the alternative distributions were selected to ensure that they produced RT distributions that were visually similar to those generated by the Wald distribution used in the main analyses. Beyond visual resemblance, particular attention was paid to approximating the key statistical moments (e.g., mean, variance, skewness) observed in the original Wald-based simulations. This approach aimed to make model comparisons as fair and interpretable as possible, given that it was not feasible to match all moments exactly by manipulating a single parameter in each distribution. Figures B1, B2, and B3 illustrate the visual alignment between distributions, and details regarding the logic of this parametrization can be found in Appendix B.

**Fitting Conditions**

The model-fitting conditions were similar to those used in Simulations 2 and 3 of the main analysis. In this case, only non-parametric models and a selected set of parametric models were estimated—specifically those assuming response times follow a shifted Wald, ex-Gaussian, shifted lognormal, or shifted gamma distribution.

Constraints on *η* were the same as those used under condition R1 (see Table 2 for an overview of how this parametrization was applied across distributions). In the unrestricted versions of both the DIM and PCRM (Model_full_), all parameters listed in Table 2 were estimated without constraints, allowing both *d* and *s* to vary freely. In the restricted versions (Model_eq_) the parameters *d* and *s* were fixed to zero. In both versions, the non-decision time parameters were held constant across the three distributional components.

**Performance Measures**

The performance measures used in C1, C2 and C3 simulations are the same as those described in Simulations 1, 2, and 3.

**Results**

**Simulation C1: Goodness of Fit**

To evaluate the goodness-of-fit test of parametric and non-parametric models under alternative data-generating distributions, we conducted a series of simulations using ex-Gaussian and lognormal distributions. This allowed us to examine whether our main conclusions, originally derived from Wald-generated data, would generalize to these empirically plausible models of RTs.

The non-parametric model consistently provided adequate fit across all simulation conditions and sample sizes, regardless of the data-generating distribution or the parameter manipulated (see Figures C1 to C4, left panels). RRates closely matched the nominal alpha level, indicating proper accommodation to the expected Type I error rate.

In contrast, the RRates in the parametric models depended strongly on the underlying assumptions. For ex-Gaussian simulated data in conditions where $\Delta\sigma$ was manipulated (Figure C1), non-true parametric models (such as Wald or Gamma) had RRate considerably higher than .05, especially when $n=300$. Conversely, in $\Delta\tau$ conditions (Figure C2), RRate remained relatively low (near .05) across all models and sample sizes. This suggests that parametric models are generally better at accommodating changes in the τ parameter than in the σ parameter of ex-Gaussian data. Note that these manipulations of τ and σ are the ones we have established as equivalent to the manipulations of location/scale and shape, respectively, for Wald data used in the main text conclusions. In the latter, we also found that in general models fit better when changing location/scale than when changing shape.

A similar pattern was observed in the lognormal simulations. When σ was manipulated (Figure C3), parametric models generally provided a poorer fit compared to the case of $\mu$ manipulations (Figure C4). However, it is worth noting that the Wald model consistently provided a much better fit to lognormal data than alternative parametric models such as ex-Gaussian or gamma. This indicates that even under σ manipulations, the Wald model performs better with lognormal data than other non-true models. Importantly, the ex-Gaussian model was unable to adequately fit lognormal data in any of the conditions examined.

To remain consistent with the main text and to simplify the interpretation of results, we continued to analyze only those models that visually showed good fit to the simulated data, that is, those that achieved RRates close to .05 as effect size increased. Among the fitted models, the gamma model provided a very poor fit to both lognormal and ex-Gaussian data. Therefore, the gamma model was excluded from subsequent analyses. For lognormal data, the ex-Gaussian model showed very poor fit and was also excluded from further model fitting with lognormal data simulations.

**Figure C1.**

*Rejection rates (RRates) for the goodness-of-fit test for the* $\Delta\sigma$ *manipulation conditions with ex-Gaussian data.*


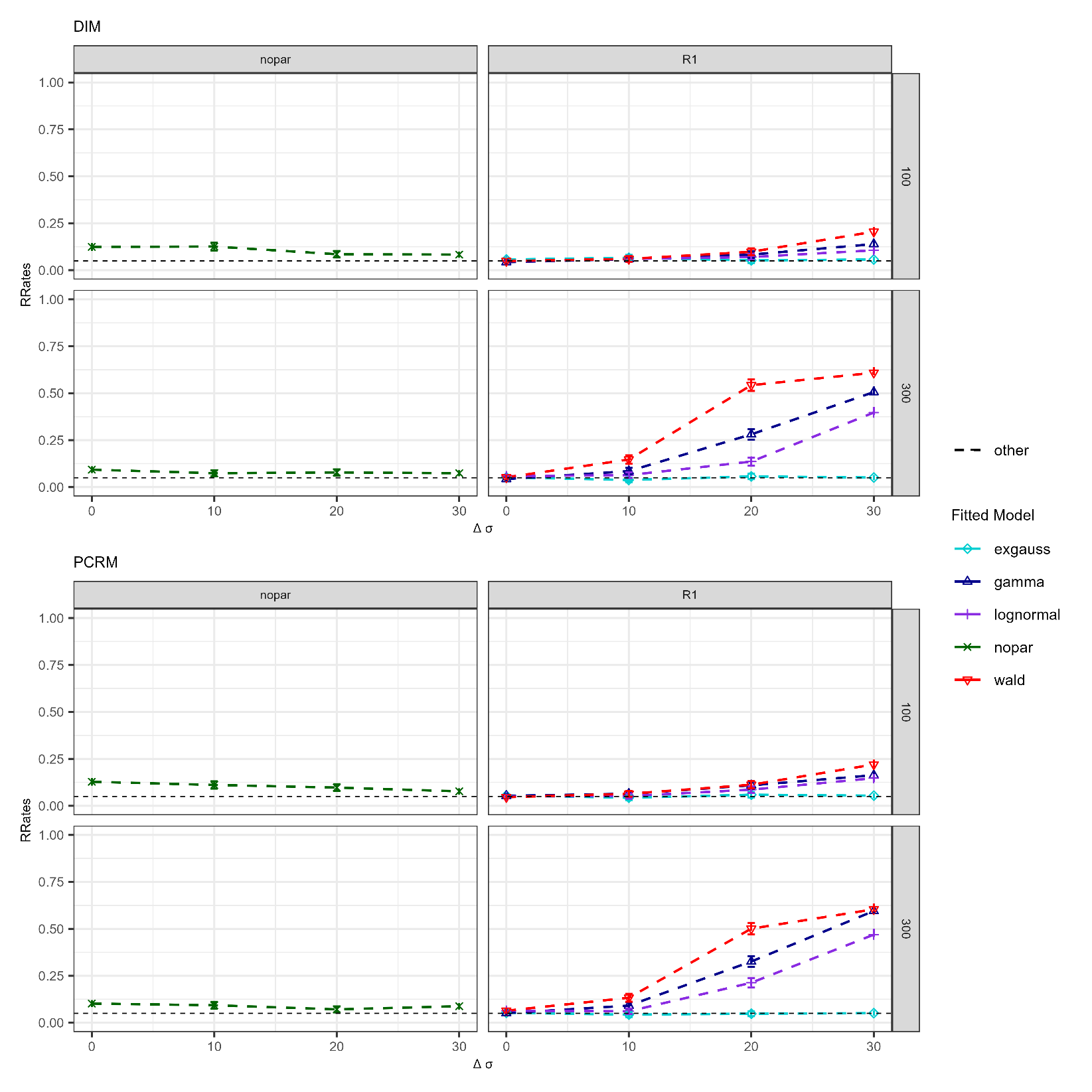


*Note*. Effect sizes were manipulated using $\Delta\sigma$ values ranging from 0 to 30 in ex-Gaussian data. DIM (top two rows) and PCRM (bottom two rows) refer to the data generation and fitting processes, respectively. The row title denotes the number of observations per tree (100 or 300), and the column title denotes the model constraint ("nopar" for non-parametric fitting and "R1" for parametric fitting with location, scale, and shape parameters allowed to vary across branches).

**Figure C2.**

*Rejection rates (RRates) for the goodness-of fit test for the* $\Delta\tau$ *manipulation conditions with ex-Gaussian data.*


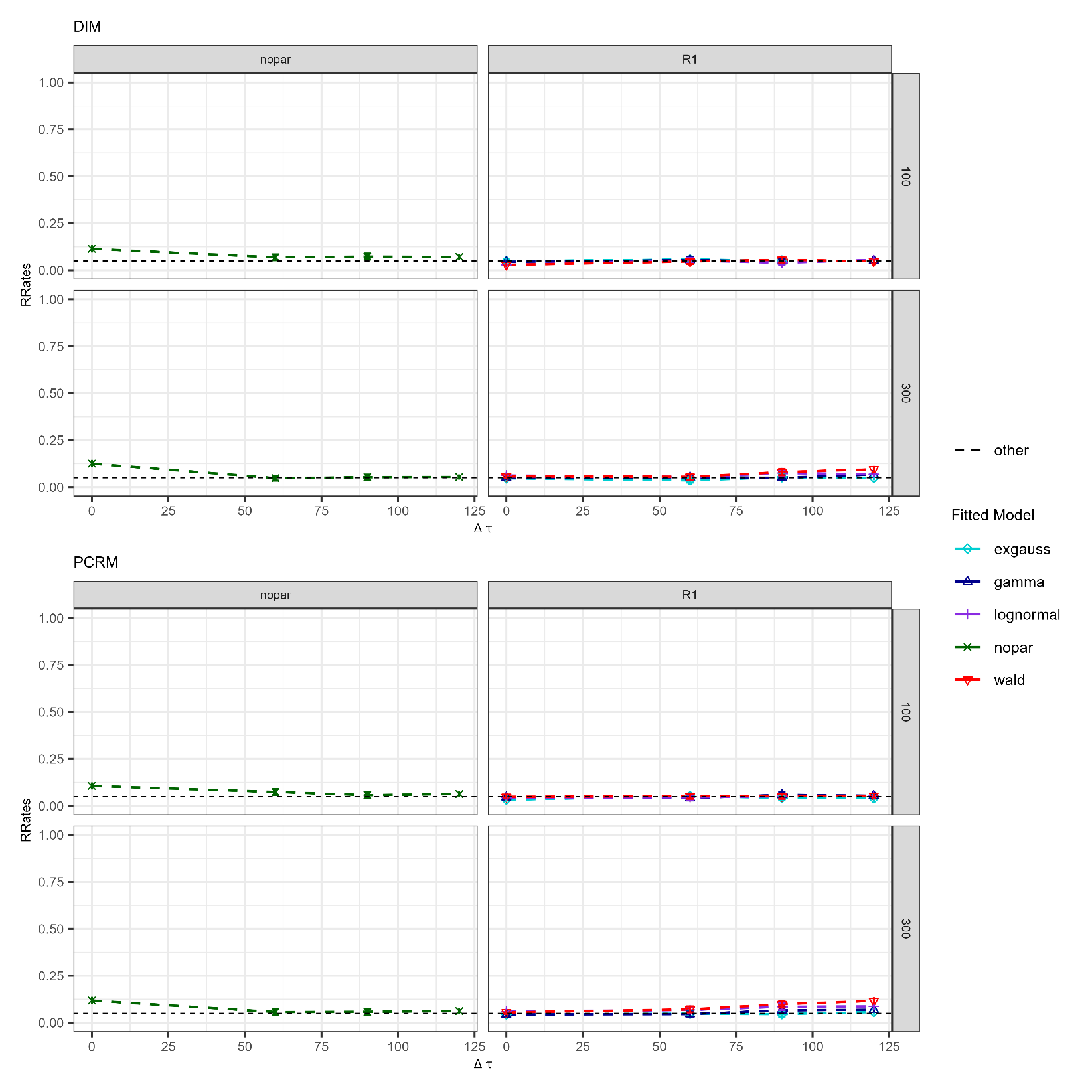


*Note*. Effect sizes were manipulated using $\Delta\tau$ values ranging from 0 to 125 in ex-Gaussian data. DIM (top two rows) and PCRM (bottom two rows) refer to the data generation and fitting processes, respectively. The row title denotes the number of observations per tree (100 or 300), and the column title denotes the model constraint ("nopar" for non-parametric fitting and "R1" for parametric fitting with location, scale, and shape parameters allowed to vary across branches).

**Figure C3.**

*Rejection rates (RRates) for the goodness-of fit test for the* $\Delta\sigma$ *manipulation conditions with lognormal data.*


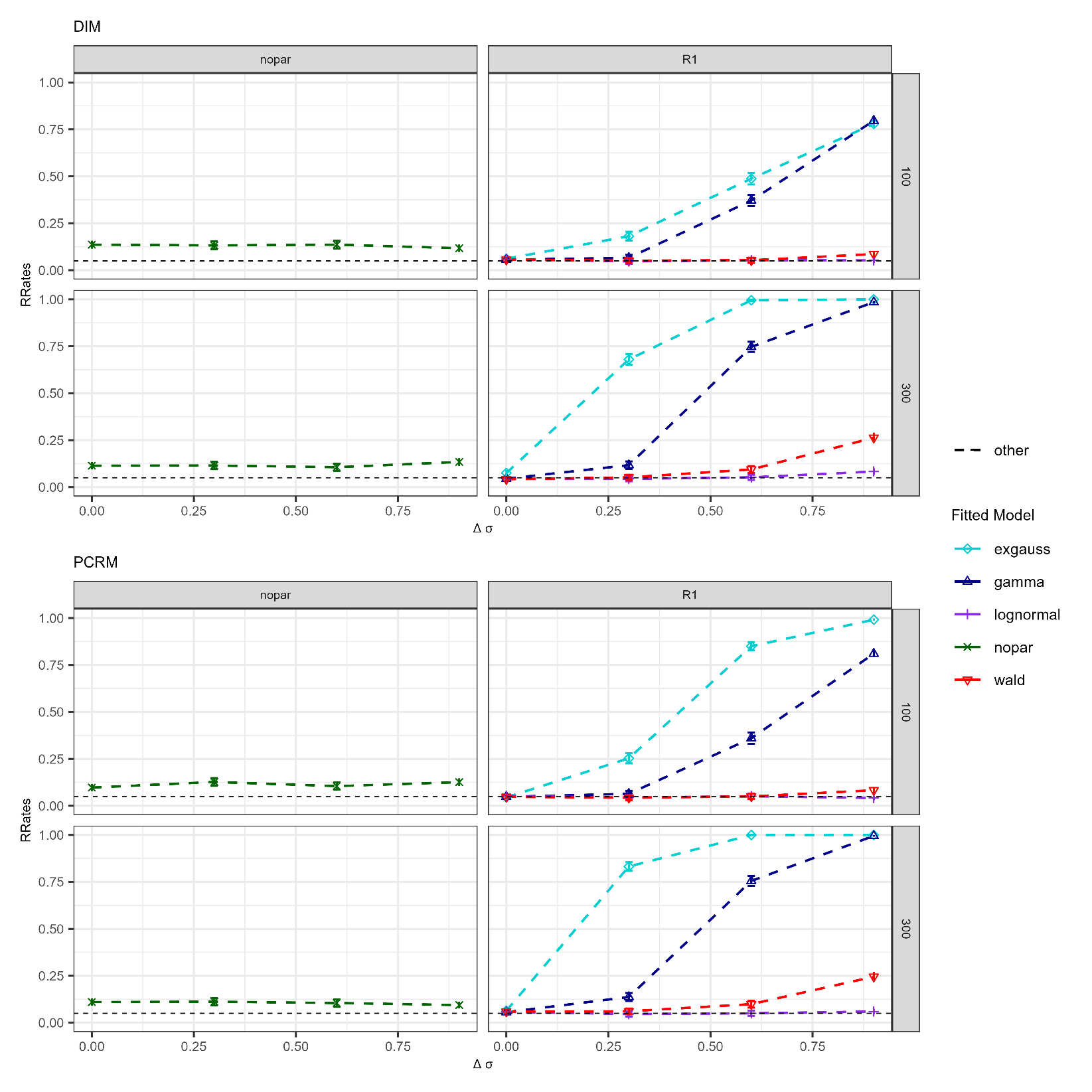


*Note*. Effect sizes were manipulated using $\Delta\sigma$ values ranging from 0 to 0.8 in shifted lognormal data. DIM (top two rows) and PCRM (bottom two rows) refer to the data generation and fitting processes, respectively. The row title denotes the number of observations per tree (100 or 300), and the column title denotes the model constraint ("nopar" for non-parametric fitting and "R1" for parametric fitting with location, scale, and shape parameters allowed to vary across branches).

**Figure C4.**

*Rejection rates (RRates) for the goodness-of fit test for the* $\Delta\mu$ *manipulation conditions with lognormal data.*

*Note*. Effect sizes were manipulated using $\Delta\mu$ values ranging from 0 to 0.8 in shifted lognormal data. DIM (top two rows) and PCRM (bottom two rows) refer to the data generation and fitting processes, respectively. The row title denotes the number of observations per tree (100 or 300), and the column title denotes the model constraint ("nopar" for non-parametric fitting and "R1" for parametric fitting with location, scale, and shape parameters allowed to vary across branches).


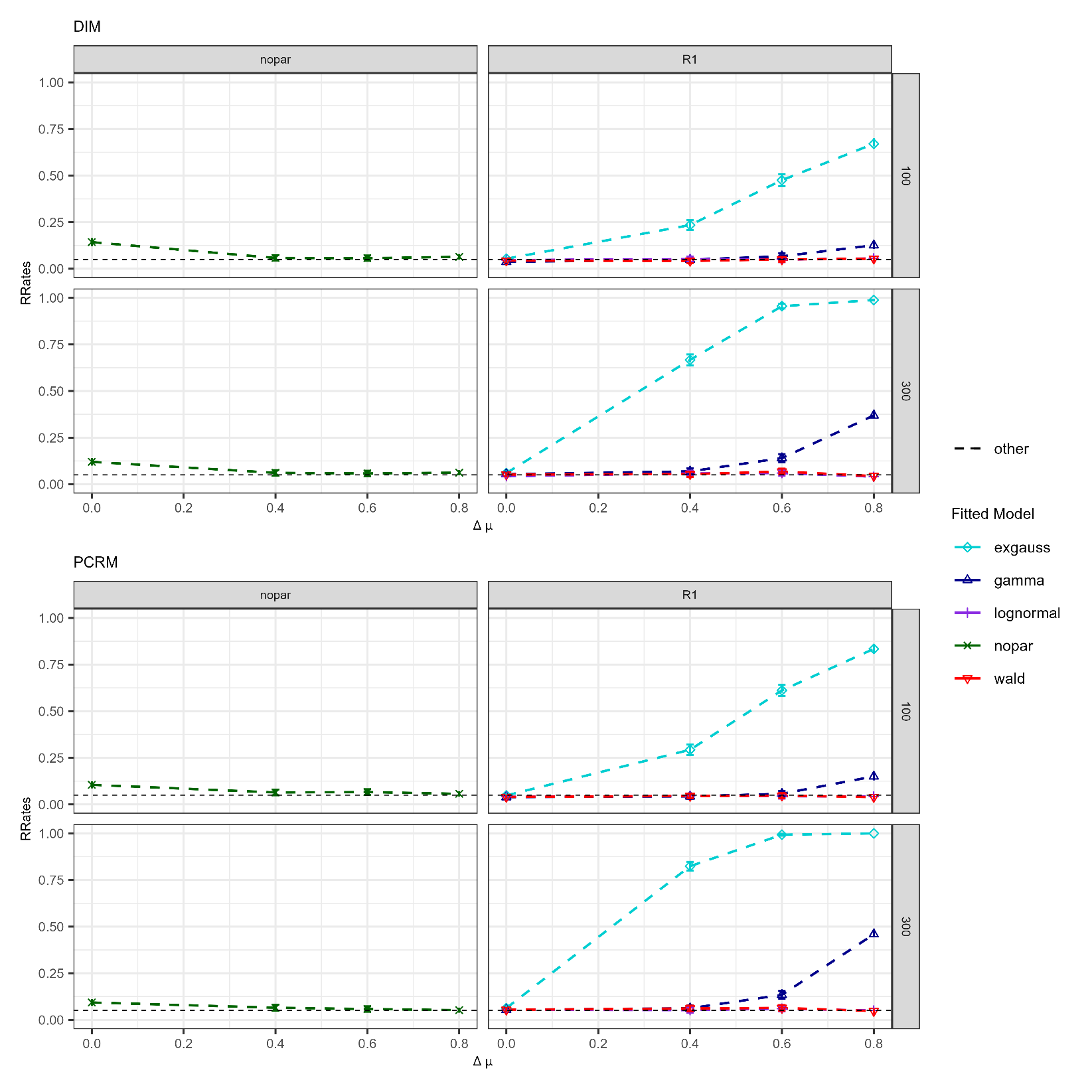


**Simulation C2: Nested Comparison**

In this section, we show RRates from the LRT, which allows us to evaluate the power to detect differences between branches within the same model, using data simulated from ex-Gaussian and lognormal models (see Figures C5–C8).

For ex-Gaussian data, we observed that non-parametric models exhibited a marked loss of power when detecting differences that stemmed from $\Delta\sigma$ manipulations. In contrast, certain parametric models, such as lognormal, demonstrated relatively higher power under these conditions. However, when the manipulation of $\Delta\tau$, both non-parametric and parametric models performed well, with RRates approaching 1.0, especially as the sample size increased ($n=300$). Under these conditions, differences between the two model types were negligible, suggesting that both approaches are equally sensitive to changes in the location/scale parameter.

For lognormal data, changes in the location/scale parameter produced high power for both non-parametric and parametric models, with little discernible difference between approaches. Notably, for shape parameter manipulations no parametric model performed comparatively well relative to the true one, whereas the loss of power for the non-parametric model was substantial.

It is important to highlight that across all conditions, the Wald model exhibits a peculiar behavior when fitted to data not generated by this distribution. Specifically, the Wald model displays a Type I error rate that is considerably higher than the nominal alpha level. In other words, it tends to detect differences that do not actually exist when the sample size is large. This inflated false positive rate raises concerns about the specificity and reliability of the Wald model in nested comparisons.

**Figure C5.**

*Rejection rates (RRates) of the nested model test with ex-Gaussian data.*


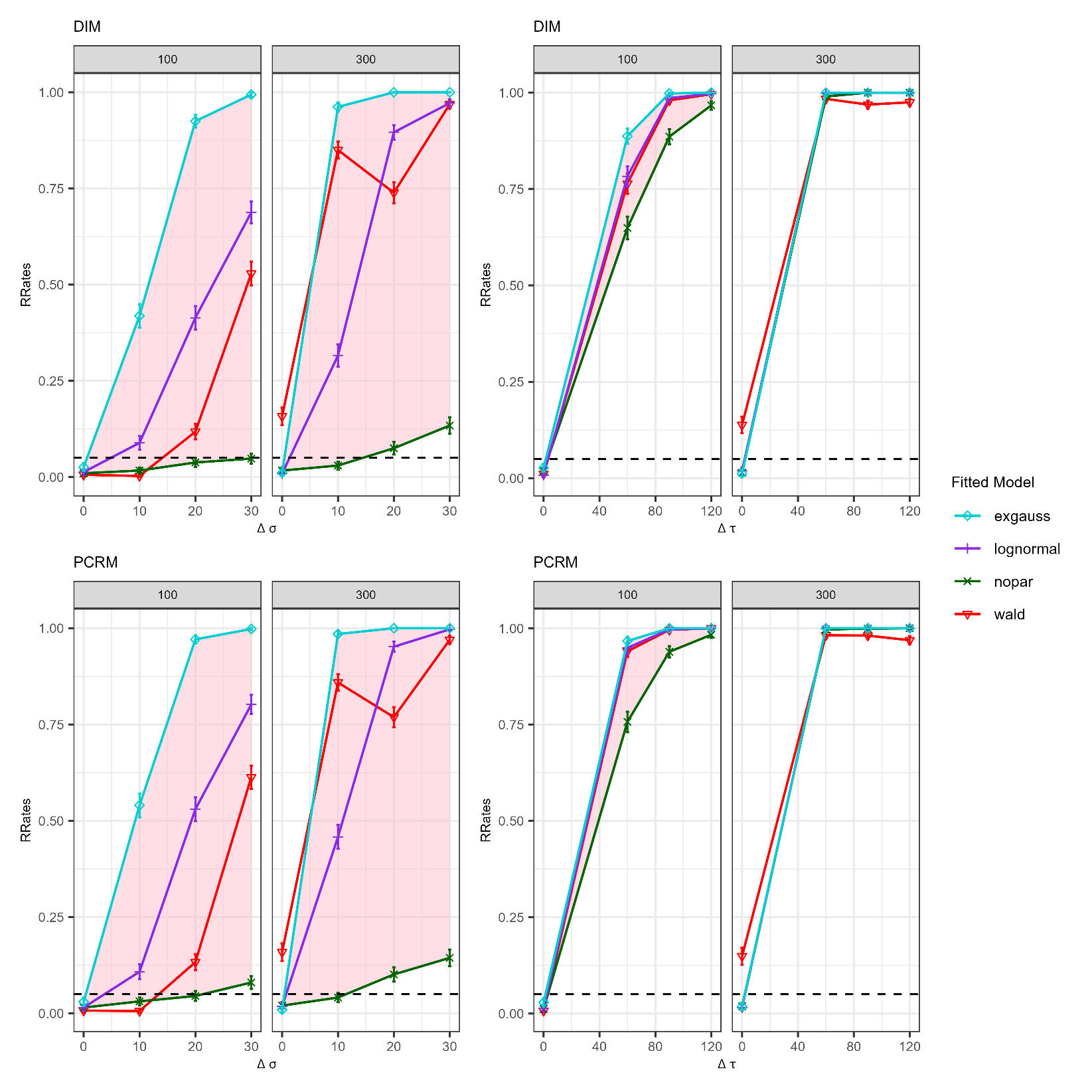


Note. Differences in ex-Gaussian distributions were manipulated via the σ parameter (left column) and the τ parameter (right column). DIM (top row) and PCRM (bottom row) refer to the data generation and fitting processes. The title of each plot denotes the number of observations per tree (100 or 300). The light red area highlights the loss in power when relying on the non-parametric model instead of the correctly specified parametric model.

**Figure C6.**

*Rejection rates (RRates) of the nested model test with shifted lognormal data*


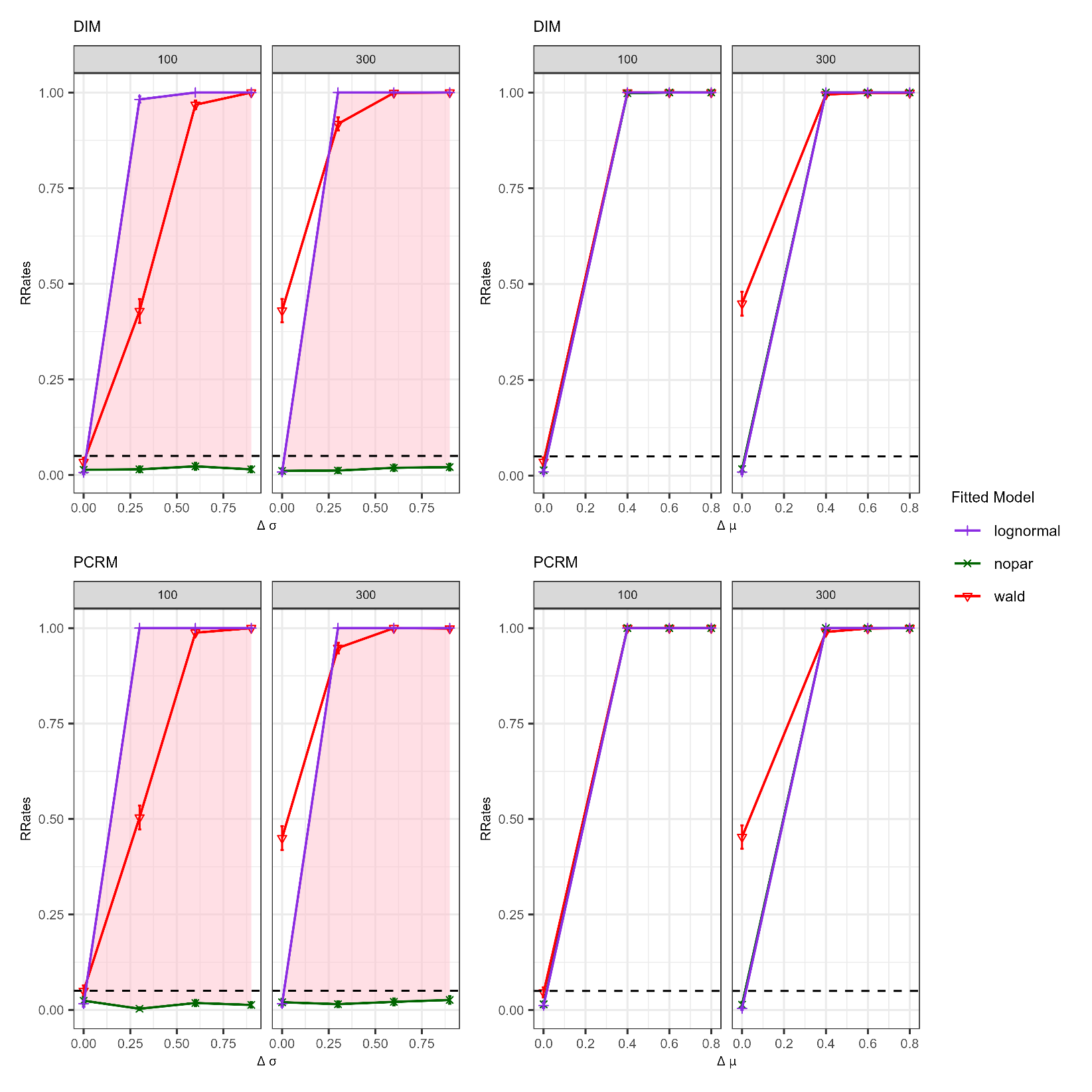


Note. Differences in shifted lognormal distributions were manipulated via the $\Delta\sigma$ parameter (left column) and the $\Delta\mu$ parameter (right column). DIM (top row) and PCRM (bottom row) refer to the data generation and fitting processes. The title of each plot denotes the number of observations per tree (100 or 300). The light red area highlights the loss in power when relying on the non-parametric model instead of the correctly specified parametric model.

**Simulation C3: Non-nested Comparison**

To evaluate the ability of parametric and non-parametric models to recover the correct model (PCRM or DIM), we present non-nested comparisons using the wAIC across data simulated with ex-Gaussian and shifted lognormal models (see Figures C7 and C8).

For ex-Gaussian data, $\Delta\sigma$ manipulations (Figure C7 left panels) revealed that non-parametric models struggled to recover the PCRM or DIM (i.e., values close to 1 for DIM and close to 0 for PCRM), while lognormal parametric models performed better under these conditions. The Wald model, which already demonstrated poor fit to ex-Gaussian data in Simulation C1, was unable to identify the true data-generating model in these scenarios. Overall, the performance of parametric models depends entirely on the family that is assumed, and which one generated the data; however, when a parametric model is well-suited to detect the specific changes present in the data, it tends to be more powerful than the non-parametric alternative. When differences involved the tau parameter (Figure C7 right panels), both parametric and non-parametric models exhibited high accuracy in model recovery. Notably, with smaller sample sizes, the parametric approach is more powerful than the non-parametric one.

A similar pattern emerged for lognormal data. The parametric Wald model tended to perform best under sigma manipulations (Figure C8 left panels), outperforming the non-parametric alternative. When differences involved $\Delta\mu$ manipulations (Figure C8 right panels), both parametric and non-parametric models again showed high accuracy in model recovery, especially with larger sample sizes where differences between approaches were negligible. In these scenarios, wAIC values strongly favored the true data-generating model, and both approaches achieved near-perfect model recovery as the effect size increased.

**Figure C7.**

*Non-nested model comparison with AIC weights in favor of the DIM relative to the PCRM for ex-Gaussian.*


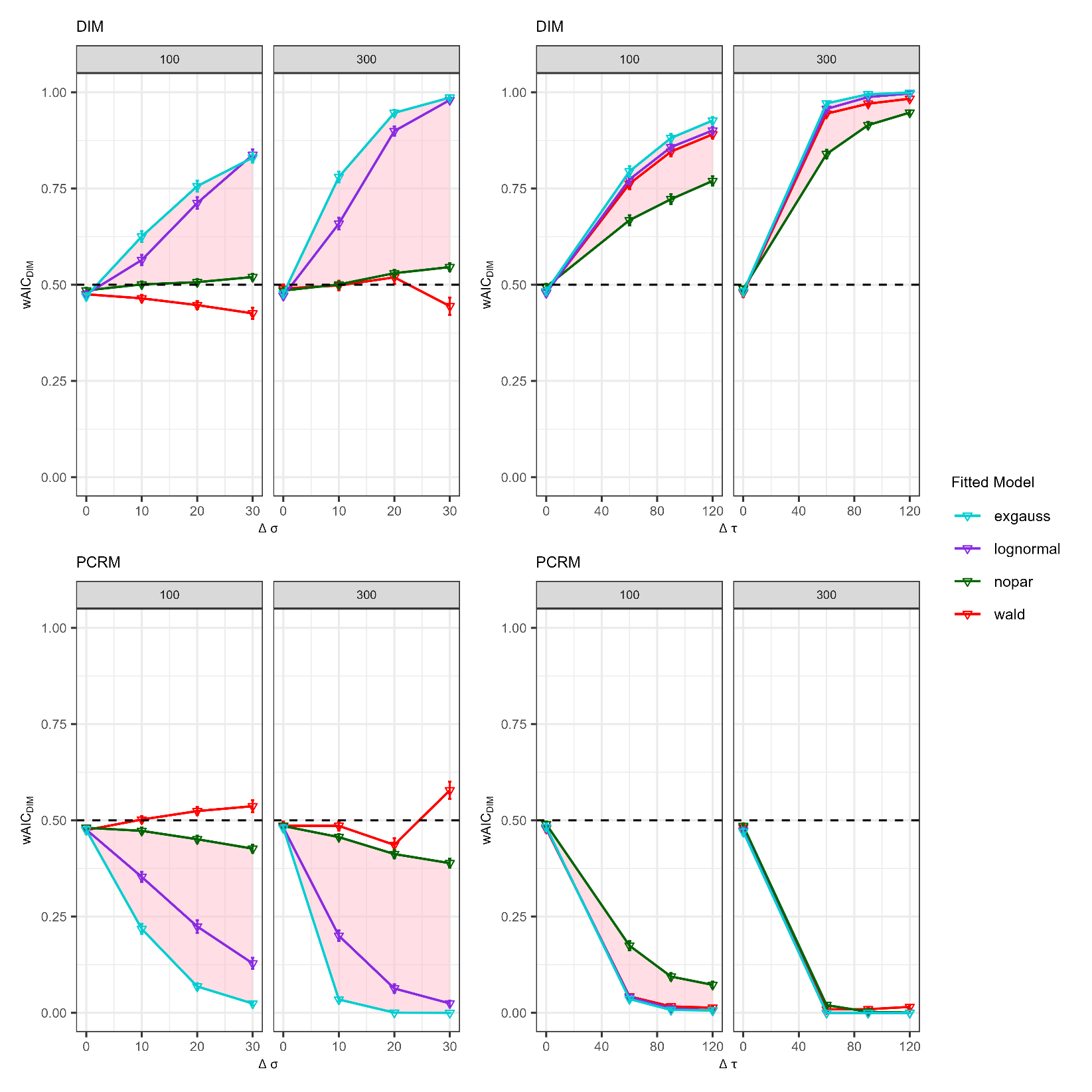


*Note*. Average $AIC$weights towards the DIM model (${wAIC}_{DIM})$ for non-nested model comparisons for ex-Gaussian data. Differences between process-specific RT distributions were manipulated via the $\Delta\sigma$ parameter (left columns) or the $\Delta\tau$ parameter (right columns). The labels DIM (top row) and PCRM (bottom row) refer to the data generating process. The title of each plot denotes the number of observations per tree (100 or 300).

**Figure C8.**

*Non-nested model comparison with AIC weights in favor of the DIM relative to the PCRM for shifted lognormal.*

*
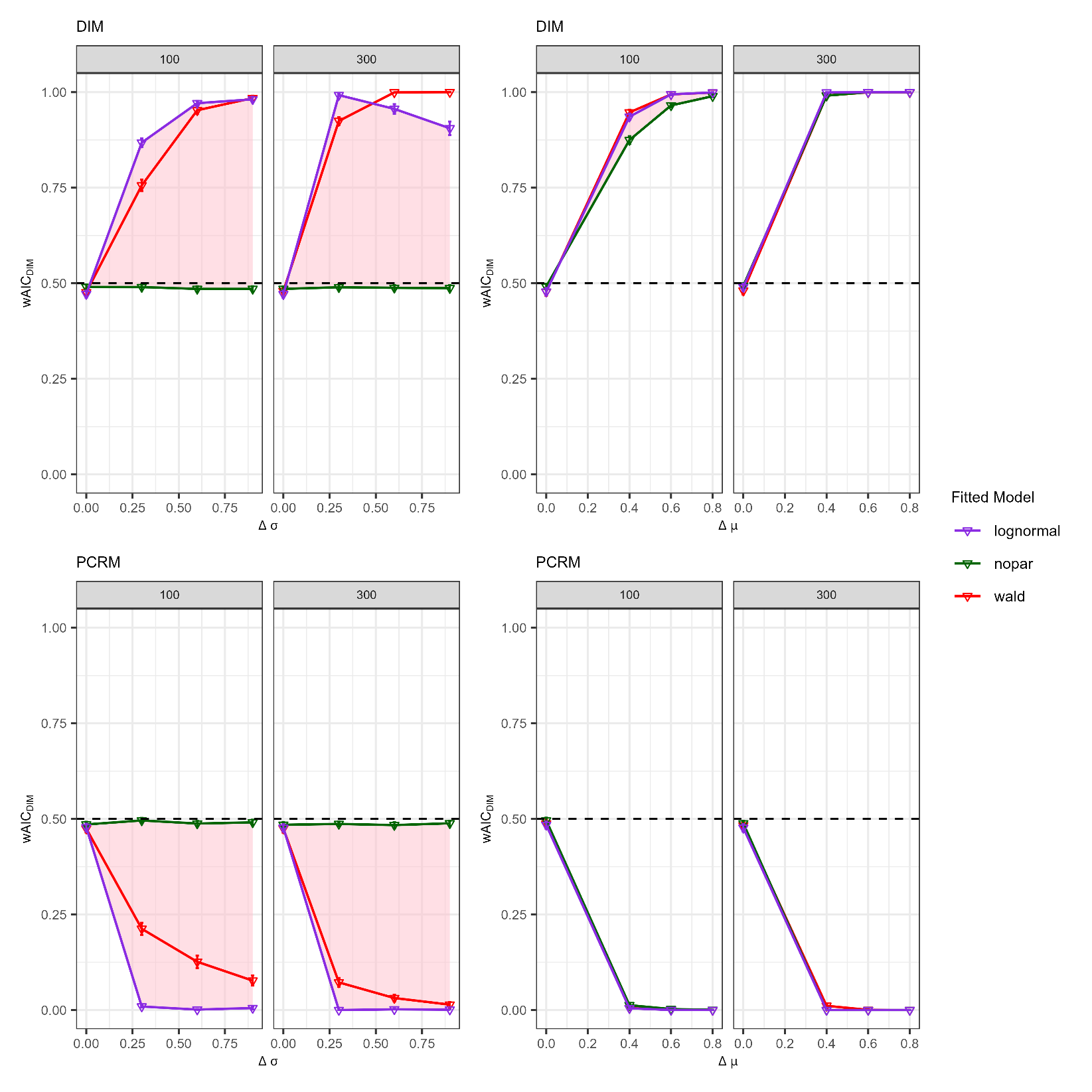
*

*Note*. Average $AIC$weights towards the DIM model (${wAIC}_{DIM})$ for non-nested model comparisons for shifted lognormal data. Differences between process-specific RT distributions were manipulated via the shape parameter $\Delta\sigma$ (left columns) or the location/scale parameter $\Delta\mu$ (right columns). The labels DIM (top row) and PCRM (bottom row) refer to the data generating process. The title of each plot denotes the number of observations per tree (100 or 300).

**Discussion**

With the simulations using lognormal and ex-Gaussian data, we aimed to establish manipulations that are equivalent to the manipulations of location/scale and shape for Wald data used in the main text conclusions. When comparing parametric and non-parametric approaches across different RT-generating distributions we observed some consistent patterns over different data-generating families.

As in Simulation 1, results in Simulation C1 show that the non-parametric approach is highly robust, consistently providing good fit across all data-generating distributions (shifted Wald, ex-Gaussian and shifted lognormal) and manipulations (shape and location/scale). In contrast, even when multiple parameters were allowed to vary, the performance of parametric models depended strongly on the assumptions of the specific distributional family chosen (e.g.: ex-Gaussian and gamma showed poor fit to lognormal data). Thus, the non-parametric models offer a reliable and assumption-free alternative, which can be advantageous when the true form of the RT distribution is unknown or there is limited theoretical knowledge about expected differences. However, this robustness comes at the cost of reduced sensitivity in several scenarios, as we have seen in Simulations C2 and C3.

In Simulation C2, we found that, as with Wald-generated data, non-parametric models exhibited substantial losses of power for ex-Gaussian and lognormal data when comparing nested models with differences driven by shape parameters. These limitations were more pronounced with smaller sample sizes. In these scenarios, certain alternative parametric models were more powerful, such as the lognormal model for detecting shape differences in ex-Gaussian data. Conversely, when differences involved location/scale parameters, both non-parametric and parametric models showed high power and near-perfect model recovery as sample size increased.

Similarly, in Simulation C3, for non-nested comparisons, we again found that the non-parametric model was much better at detecting location/scale manipulations in lognormal and ex-Gaussian data than shape differences. For subtle shape differences, parametric models may be a better option. For example, the lognormal model can detect shape differences in ex-Gaussian data with relatively little loss of power compared to the true data-generating model, and the Wald model can do so with lognormal data when shape is manipulated.

Overall, the non-parametric approach offers a safeguard against model misspecification but may lack sensitivity for detecting certain effects, such as subtle shape differences. Parametric models, on the other hand, can provide higher sensitivity and power—but only when their assumptions closely match the underlying data distribution. In summary, our results underscore the importance of weighing the trade-offs between robustness and sensitivity by considering the likely nature of the data distribution, our theoretical predictions, and the strength of those predictions.

1. The supplementary material includes the scripts that calculate the moments for each distribution based on changes in each parameter. [↑](#footnote-ref-1)
